# A Statistical Analysis of Risk Factors and Biological Behavior in Canine Mammary Tumors: A Multicenter Study

**DOI:** 10.3390/ani10091687

**Published:** 2020-09-18

**Authors:** Giovanni P. Burrai, Andrea Gabrieli, Valentina Moccia, Valentina Zappulli, Ilaria Porcellato, Chiara Brachelente, Salvatore Pirino, Marta Polinas, Elisabetta Antuofermo

**Affiliations:** 1 Department of Veterinary Medicine, University of Sassari, Via Vienna 2, 07100 Sassari, Italy; gburrai@uniss.it (G.P.B.); agabrieli@uniss.it (A.G.); pirino@uniss.it (S.P.); mpolinas@uniss.it (M.P.); 2 Mediterranean Center for Disease Control, University of Sassari, Via Vienna 2, 07100 Sassari, Italy; 3 Department of Comparative Biomedicine and Food Science, University of Padova, AGRIPOLIS–Viale dell’Università 16, 35020 Legnaro Padua, Italy; valentinamoccia1993@gmail.com (V.M.); valentina.zappulli@unipd.it (V.Z.); 4 Department of Veterinary Medicine, University of Perugia, 06123 Perugia, Italy; ilariaporcellatodvm@gmail.com (I.P.); chiara.brachelente@unipg.it (C.B.)

**Keywords:** age, breed, mammary tumor size, dogs, machine learning, reproductive and hormonal status

## Abstract

**Simple Summary:**

The increase in the incidence of neoplastic disease represents a relentless challenge in veterinary medicine, and many efforts aimed to increase early diagnosis and life perspective have been made. Canine mammary tumors are the most common neoplasm and one of the leading causes of death in female dogs. Using a large number of data from three academic institutions, we found that dogs with malignant tumors were significantly older than dogs harboring benign tumors and that malignant tumors were significantly larger than benign counterparts. Moreover, a consistent fraction of malignant tumors is smaller than 1 cm, providing compelling evidence that the size of mammary tumors is a critical but easily detectable, indirect prognostic-related, clinical factor. We suggest that the control of cancer-related risk factors represents one of the most compelling prevention strategies and paves the way for further investigations.

**Abstract:**

Canine mammary tumors (CMTs) represent a serious issue in worldwide veterinary practice and several risk factors are variably implicated in the biology of CMTs. The present study examines the relationship between risk factors and histological diagnosis of a large CMT dataset from three academic institutions by classical statistical analysis and supervised machine learning methods. Epidemiological, clinical, and histopathological data of 1866 CMTs were included. Dogs with malignant tumors were significantly older than dogs with benign tumors (9.6 versus 8.7 years, *p* < 0.001). Malignant tumors were significantly larger than benign counterparts (2.69 versus 1.7 cm, *p* < 0.001). Interestingly, 18% of malignant tumors were smaller than 1 cm in diameter, providing compelling evidence that the size of the tumor should be reconsidered during the assessment of the TNM-WHO clinical staging. The application of the logistic regression and the machine learning model identified the age and the tumor’s size as the best predictors with an overall diagnostic accuracy of 0.63, suggesting that these risk factors are sufficient but not exhaustive indicators of the malignancy of CMTs. This multicenter study increases the general knowledge of the main epidemiologica-clinical risk factors involved in the onset of CMTs and paves the way for further investigations of these factors in association with CMTs and in the application of machine learning technology.

## 1. Introduction

Cancer is the leading cause of death in companion animals, and mammary tumors, the most common neoplasm in female dogs, represents a serious issue in worldwide veterinary practice [1,2]. As in animals, human breast cancer (HBC) is the most common malignancy in women worldwide, and several clinical and molecular similarities between canine mammary lesions and HBC have been described [3,4]. Consequently, dogs have attracted considerable interest as potential animal models to study human cancer [5]. Despite the tremendous effort made in fighting cancer, the biological and morphological heterogeneity of canine mammary tumors (CMTs) has challenged veterinary pathologists since the early days of diagnostic pathology. As a result, an increasing number of studies in this area have been published in recent decades. Mammary cancer is a multifactorial disease and various elements contribute to its occurrence and behavior [6].

Epidemiological, clinical, histological, and molecular factors are considered important risk factors for mammary neoplasms. Among the main epidemiological CMT risk factors, age, breed, and reproductive and hormonal status are consistently reported in the literature [1,2,6].

CMTs usually affects middle-aged and older dogs with an increased risk between 8–11 years old [6]. Additionally, benign tumors are more likely in dogs ranging between 7 and 9 years, while malignant tumors are more frequently encountered in older dogs [7,8,9,10,11]. However, the peak incidence dictated by the age should be carefully evaluated given that larger breeds of dogs have a naturally shorter lifespan and therefore tend to be younger than smaller breeds when they receive a cancer diagnosis [1].

Mammary neoplasm can occur in dogs of any breed, although pure breeds seem more prone to develop CMTs [1,2,6,10]. Poodles, Chihuahuas, Dachshunds, Yorkshire Terriers, Maltese, and Cocker Spaniels are frequently listed as high-risk dog breeds in the small breed category. Some of the larger breeds are also at higher risk, including the English Springer Spaniel, English Setters, Brittany Spaniels, German Shepherds, Pointers, Doberman Pinschers, and Boxers [1,2,6,10,11]. However, considerable discrepancies exist between studies regarding the breed as a CMT risk factor. A representative case is the evaluation of familial or inherited germline mutations in Breast Cancer 1 and 2 genes (BRCA1 and BRCA2), that in women are related to an increased lifetime risk of HBC, but that led to nonspecific results in veterinary medicine [12,13,14,15].

More consistent data are reported regarding the sex hormones effect, with a general agreement on the concept that the exposure to endogenous ovarian hormones is a cause of mammary tumor development in dogs [1,2] and references therein. According to Schneider and colleagues in 1969 [16], dogs spayed before their first estrus have a 0.5% risk of developing CMTs in their lifetime, while the benefits of the ovariohysterectomy diminish with each estrus cycle. It seems biologically plausible to state that the greatest benefit on CMTs prevention is exerted when the dogs are spayed early in their reproductive lifetime, probably by reducing the occurrence of proliferation stimuli and therefore the risk for cancer-related events (e.g., mutations) [3,4,17]. Furthermore, a more recent prospective randomized study reported a significantly decreased risk for new tumor development by performing ovariohysterectomy concurrently with benign CMT removal [18], while the same effect was not observed for malignant tumors [19].

Clinical features of CMTs are also considered as prognostic factors by numerous studies [1,20,21,22,23,24]. Of note, the tumor size (T), the involvement of lymph nodes (N), and the presence of distant metastasis (M) are the key features of the clinical, prognosis-related, “TNM” staging system, developed in 1980 by the World Health Organization (WHO) and recently revised [1,20,21,22,23,24].

In the recent WHO version [1], stage advances from I to II to III as the size of the primary tumor increases from smaller than 3 cm, to between 3 and 5 cm, to larger than 5 cm. Lymph node metastasis represents stage IV disease, regardless of tumor size, and distant metastasis constitutes stage V. Notably, the size of the tumors represents a critical parameter in stage I, II, and III and strongly impacts on CMT prognosis and outcome. According to MacEwen et al. [25], 1985, dogs with tumors larger than 3.4 cm in diameter have a statistically significant worse outcome than dogs with smaller tumors, both in terms of remission and survival. Other authors, however, have found a change in prognosis only when tumors are larger than 5 cm [21]. In one study, tumor size was not prognostic when node involvement was detected [24]. Despite these studies, the importance of the tumor size is a biologically trustworthy factor, considering that more aggressive tumors grow faster and, therefore, are larger and more likely to harbor metastatic subclones [8]. Hence, the staging systems integrating different clinical parameters provide specific recommendations to clinician’s treatment decision making [1,26,27].

In this study, we evaluated in a large retrospective statistical analysis the breed, the spayed status, and the age as epidemiological risk factors and the tumor size as a clinical prognostic-related feature of 1866 CMTs collected from three different Departments of Veterinary Medicine of the University of Sassari (UNISS), Padua (UNIPD), and Perugia (UNIPG). We analyzed the relationship between some epidemiological-clinical risk factors and the histological diagnosis to test the ability to prompt clinical data in predicting the diagnosis and, indirectly, a prognostic outcome. A supervised machine learning technique was compared to the classical statistical analysis and used to investigate the ability to predict the diagnosis of CMTs (malignant versus benign).

## 2. Materials and Methods

This retrospective study focused on reviewing CMT data generated from 3 different tumors databases (UNISS, UNIPD, UNIPG). Experiment permission was not required from the University’s Animal Care Ethics Committee because all the samples were retrieved from the archive of the pathology laboratories and were used for diagnostic purposes.

The inclusion criteria for data selection were: dogs with single mammary neoplasia, availability of documented medical history including breed, age, macroscopical tumor size as indicated either by the clinician or by the histological laboratory and histopathological diagnosis of the neoplasm.

All previous histological diagnoses were updated and classified according to the recent publication of Surgical Pathology of Tumors of Domestic Animals, Volume 2: Mammary tumors [28].

### 2.1. Statistical Analysis—Descriptive Statistics and Univariate Analysis

To determine whether there was an association between epidemiological (age, breed, spayed status) and clinical characteristics (tumor size) and tumor diagnosis, the breed, age, spayed status, and tumor size were examined in association with the histological diagnosis. For statistical purposes, the breed was classified as pure breed and mixed breed, the age was either treated as a numerical variable (in years) or categorized in 4 classes (0–4 years; 5–8 years; 9–12 years and >13 years).

The greatest diameter of the tumor (i.e., the size) was treated as a numerical variable (in centimeters), or categorized according to WHO TNM system (3 categories: T1 < 3 cm, T2 = 3–5 cm, and T3 > 5 cm) or as proposed by Sonremno and colleagues (5 categories: S1 < 1 cm, S2 = 1 to <2 cm, S3 = 2 to <3 cm, S4 = 3 to <5 cm, S5 > 5 cm) [1,8].

According to Pena et al., 2013, and references therein, malignant tumors were grouped into 3 histological categories (i.e., HD3 categories) based on morphological features and biological behavior as follows: group I, which included in situ carcinoma, simple carcinoma, carcinoma arising in a mixed tumor, complex carcinoma, mixed-type carcinoma, ductal carcinoma, and adenosquamous carcinoma; group II, which included solid carcinoma, comedocarcinoma, carcinoma, and malignant myoepithelioma, and anaplastic carcinoma; group III, which included other histological types [24].

Statistical analysis was carried out using a Student’s T test for continuous normally distributed variables, chi-square (X^2^) test and nonparametric Kruskal–Wallis ANOVA followed by Dunn’s post hoc test for categories. Data were analyzed with Stata version 11.2 (StataCorp, 2009), and results were considered significant when *p* ≤ 0.05.

### 2.2. Statistical Analysis—Multivariate Analysis and Machine Learning Model

Logistic regression analysis was performed to evaluate the influence of the different covariates (age, tumor size, spayed status, and breed) on tumor diagnosis. Covariates were selected through a nested likelihood ratio test (Table 1 and Supplementary Materials).

The selected continuous covariates were then converted into categorical covariates according to the previously described schemes, generating two further models: the IC model where the tumor size was encoded according to the WHO TNM system and the IIC model where the tumor size was split into 5 categories as previously reported by Sonremno et al., 2009 [1,8].

Machine learning was performed to investigate the possibility to predict the diagnosis of mammary neoplasms in the dog (malignant versus benign) based on the recorded epidemiological (breed, spayed status, and the age) and clinical (tumor size) factors. Models were built using the R programming language relying upon the caret package through algorithms provided by the GLM (for logistic regression), and the GBM (for stochastic gradient boosting) libraries [29,30,31,32,33,34,35] (see Supplementary Materials for details). In particular, the supervised machine learning technique employed is stochastic gradient boosting which is a powerful learning method based on the combination of many simple models. The basic idea is to apply sequentially a “weak” learner (here, a decision tree) to modified versions of the initial data. Each time a tree is built, the data are modified by applying weights to increase the influence of misclassified observations. The final classification is performed through a weighted majority vote [36,37,38,39]. To assess the predictive performances of logistic regressions (GLM) and stochastic gradient boosting (GBM), a nested cross-validation was performed [39]. The dataset was split into 5 nonoverlapping training and a test sets by keeping 80% of cases for training. The split was performed randomly within each of the two classes of the outcome, to preserve the overall class distribution of the data. For each of the two classifiers (even if not required for GLM, using the same procedure allows for an easier comparison), the tuning of the hyperparameters was performed through 10-fold cross-validation repeated 5 times [34,36]. Continuous features were centered and scaled. The best setup was chosen by optimizing the area under the receiver operating characteristic (ROC) curve [40] and, with such parameters, a final fit was performed on the entire training set. The final result was obtained by repeating the procedure for each outer split and taking the average over the test sets.

## 3. Results

### 3.1. Descriptive Data and Histological Diagnosis

The databases account for 1866 single mammary neoplasms. According to the histological classification, 867/1866 (46.5%) were benign tumors (BTs) and 999/1866 (53.5%) malignant tumors (MTs). According to the applied classification [28], 239/1866 (12.8%) were diagnosed as simple benign tumors, 628/1866 (33.6%) as nonsimple benign tumors, 624/1866 (33.4%) as simple malignant, 341/1866 (18.27%) as nonsimple malignant, 25/1866 (1.34%) as special type malignant and 9/1866 (0.5%) as sarcomas. Specific histological diagnoses are reported in Table 2. The most frequent benign lesions were benign mixed tumors (329/867; 37.95%) followed by complex adenomas (281/867; 32.41%) and simple adenomas (225/867; 25.9%) while the most frequent malignant tumors were simple tubulopapillary (256/999; 25.6%) followed by complex carcinomas (248/999; 24.92%). Additionally, according to Pena et al., 2013, 837/999 (83.7%) of malignant tumors were included in group I, 148/999 (14.81) in group II and 14/999 (1.4%) in group III [24].

### 3.2. Descriptive Statistics and Univariate Analysis: Breed, Age, Spayed Status, Tumor Size and Their Association with the Presence of Canine Mammary Tumors

#### 3.2.1. Breed

Sixty-one percent (1142/1866) of CMTs were observed in pure breed dogs, mostly represented by small size dogs, while the other 39% (724/1866) were observed in mixed breed dogs.

Although BTs occur predominantly in small breed dogs with Yorkshire terrier breed the most represented (64/867; 7%) and MTs occur mostly in German Shepherd dogs (79/999; 7.91%), no statistically significant association was observed between breeds and the prevalence of BTs and MTs. Similar results were noticed for the three histological malignant categories proposed by Pena (X^2^ (2) = 0.9090, *p* = 0.635).

#### 3.2.2. Age

The mean age of dogs with mammary tumors was 9.20 ± 2.63 years (mean ± SD). Furthermore, dogs with malignant mammary neoplasm were older (n: 999; mean 9.61 ± SD 2.63 years) compared with those harboring benign tumors (n: 867; mean 8.74 ± SD 2.54) (T test = −7.2679, *P* < 0.001). The studied population was divided into four age groups (0–4 years; 5–8 years; 9–12 years; 13–22 years). Interestingly, 1587/1866 (85%) of CMT were found between 5–12 years with an increased prevalence of BTs and MTs in the 9–12 age range (918/1866; 49.20%) (X^2^ (3) = 52.2150; *p* < 0.001).

Moreover, both benign tumors, either simple and nonsimple, occur at a younger median age (9 years old) than simple malignant (median = 10 years old) and malignant special type (median = 11 years old), while simple malignant occurs at an older age than nonsimple malignant (median = 11 years old) (Kruskal–Wallis χ^2^ (corrected for ties) = 68.471, *p* < 0.001; Dunn’s post hoc test, *p* < 0.001).

Noteworthy, no statistically significant difference was recorded based on the three histological malignant categories proposed by Pena et al., 2013 (Kruskal–Wallis χ^2^ (corrected for ties) = 3.870, *p* = 0.14) [24].

#### 3.2.3. Spayed Status

In our study, 1556/1866 (83.39%) tumors were diagnosed in nonspayed dogs, of which 47.04% (732/1556) were benign and 52.96% (824/1556) malignant tumors (X^2^ (1) = 1.2696; *p* = 0.260). No significant association was recorded between the spayed status and the three histological categories proposed by Pena and colleagues (X^2^ (2) = 0.3241; *p* = 0.850) [24].

However, a high prevalence of nonsimple benign tumors (34.51%; 537/1556) were diagnosed in nonspayed dogs, while in spayed dogs 39.35% (122/310) of tumors were simple malignant type (X^2^ (5) = 12.4204; *p* = 0.029).

#### 3.2.4. Tumor Size

The tumor size is considered among the most clinical prognostic-related feature. The tumor size ranged from 0.1 to 30 cm (n = 1866; mean = 2.23 ± SD 2.39 cm) and benign tumors were smaller (n: 867; mean 1.70 ± SD 1.97 cm) than malignant ones (n: 999; mean 2.699 ± SD 2.62) (T test = −9.0967; *p* < 0.001). Based on the three histological categories proposed by Pena [24], grade I tumors had a lower diameter (n = 837; mean 2.56 ± SD 2.62, median 2, ranges 0.2–30 cm) compared to both grade II (n = 148; mean 3.19 ± SD 2.40, median 3, ranges 0.3–13 cm) and grade III (n = 14; mean 4.77 ± SD 3.17, median 3.7, ranges 1–10 cm) (Kruskal–Wallis χ^2^ (corrected for ties) = 26.820, *p* < 0.001; Dunn’s post hoc test, *p* < 0.001).

According to the WHO tumor size system [1], there were 1363/1866 (73%) T1 neoplasms (<3 cm), 310/1866 (16.6%) T2 neoplasms (3–5 cm), and 193/1866 (10.3%) T3 neoplasms (>5 cm). Interestingly, 89.65% (1673/1866) of the CMTs were found between 0–5 cm and, remarkably, 63.76% (637/999) of the malignant tumors were less than 3 cm (T1 class), of which 398/637 (62.5%) were classified as simple carcinoma. T2 and T3 classes included 21.32% (213/999) and 14.91% (149/999) of the MTs.

Considering the high percentage of tumors within the range limit 0 to 5 cm, a further new variable of tumor size with five categories subclassification was applied. The S2 category (less than 2 cm in diameter) included 68.5% of benign neoplasms, whereas 63.76% of MTs were smaller than 3 cm (X^2^ (4) = 128.0751; *p* < 0.001). Interestingly, 17.72% (177/999), 27.13% (271/999), and 18.92% (189/999) of these MTs were observed in the S1 (<1 cm), S2 (from 1 to 2 cm) and S3 (from 2 to 3 cm) class, respectively, with a higher percentage of simple carcinomas compared to complex ones.

### 3.3. Multivariate Analysis and Machine Learning Model

According to the likelihood ratio and Wald test performed on the logistic regression, the tumor size and the dog’s age were significantly related to the histological diagnosis, differently than what was observed for the spay status and breed (Table 1).

Given the values of the exponentiated coefficients and using the age and tumor size covariates as continuous variables (model II), a 25% increase in the odds of a malignant tumor per 1 cm increase in tumor size adjusting for age was observed. Similarly, the logistic regression model estimates a 12% increase in the odds of a malignant tumor per 1 year increase in age, adjusting for tumor size.

Furthermore, when continuous variables were converted in categorical covariates (Table 3), a 2.3- and a 3.6-fold increase in the odds of a malignant tumor was observed when passing from T1 (<3 cm) to T2 (from 3 to 5 cm), and from T1(<3 cm) to T3 (<5 cm), respectively (*p* < 0.05).

A similar pattern is present for the IIC model; compared to the reference level (0–1 cm) the odds ratios of all other tumor size groups were larger than 1, progressing from 1.3 (tumor size 1 to 2 cm) to 4.9 in neoplasm larger than 5 cm (Table 3). In both models, only animals with an age greater than 12 years have more than a 2-fold increase in the odds of an MT when compared to the baseline age of 0–4 years.

Predictive performances of the logistic regression in terms of overall accuracy, positive predictive values (PPVs), and negative predictive values (NPVs) (i.e., number of malignant -PPV- and benign -NPV- tumors correctly diagnosed) were 0.63 (CI 0.60–0.65), 0.65 (CI 0.63–0.67), and 0.61 (CI 0.57–0.64), respectively. The GBM machine learning model had a similar predictive performance compared to the logistic model (Table 4), probably as a consequence of the small number of predictors of the dataset, which does not allow for the full exploitation such a technique to model complex nonlinear relationships possibly present in the data [41,42]. Interestingly, the tumor size and the age had a relative influence of ~69% and ~30%, respectively, while the breed (<1%) and the spay status (<1%) were insignificant in the gradient boosting model. R code, and corresponding output can be found in the Supplementary Material.

## 4. Discussion

In veterinary medicine, the increase in the incidence of neoplastic disease represents a relentless challenge for veterinary oncology specialists. Consequently, many efforts have been made in the on-going research to increase the early diagnosis and life perspective in dogs harboring mammary tumors. As a consequence, in this background, cancer research is mainly focused on the discovery and control of cancer-related risk factors [43,44]. However, a large retrospective statistical analysis that related the breed, hormonal status, age, and tumor size with the histological diagnosis and, consequently, with the possible behavior of CMTs, has not been previously performed.

In this work, an approximately equal proportion of benign (46.5%) and malignant tumors (53.5%) was observed, and mixed BTs accounted for the highest number of the total cases. Mixed neoplasms are the most frequent neoplasias in female dogs, and are characterized by the proliferation of both luminal epithelial and interstitial myoepithelial elements admixed with foci of mesenchymal tissues such as cartilage, bone, and fat [28,45]. The most frequent MT was simple tubular or tubulopapillary carcinoma (26.1%) followed by complex carcinoma (13.3%) confirming what has been reported in the literature [10] and references therein.

In our study, sixty-one percent of CMTs were observed in pure breed dogs, suggesting, as previously described by Sorenmo and colleagues [6], that the breed could be a putative risk factor, and that certain breeds, such as Miniature Toy, Shih Tzu as well as German Shepherd, are prone to develop mammary neoplasms [1,2,6,10,11]. Interestingly, in our study, benign tumors occurred predominantly in small breed dogs, particularly in Yorkshire terriers, while malignant ones were detected with higher frequency in German Shepherd dogs. A better prognosis for small breeds has been previously reported in a retrospective multivariate survival analysis [46]. However, given the increasing prevalence of CMTs in small breeds, it is uncertain whether small size in dogs could represent a reliable risk factor or if these data are influenced by the greater veterinary care in those breeds than larger dogs [10]. According to Salas and collaborators [7], no significant association was observed between the breed and the development of BTs, MTs, as well as with the malignant carcinoma categories proposed by Pena et al., 2013 [24]. Similarly, the breed showed a slight influence in the logistic and GBM machine learning models (<1%), corroborating the considerable divergences between studies regarding the breed as a CMT risk factor. Moreover, considering that the mutations in Breast BRCA1 and 2 genes and their protein products have been variably associated with the development of CMTs, a definitive conclusion about CMT breed-related risk should be performed in the context of genetic research [12,13,14,15].

Age is considered one of the most important risk factors for developing mammary tumors with a peak incidence between 8 to 11 years, with younger dogs prone to having BTs [6,7,8,9]. These data seem to be confirmed by our study, in full agreement with what was reported by Sorenmo [6].

Noteworthy, simple MTs occurred at an older age than nonsimple ones. According to different authors [47,48], simple carcinomas have a poor prognosis compared to complex ones confirming, as proposed by Pena and collaborators [24], that the age should be considered an indirect, but a strong, prognostic factor. Furthermore, these data are supported by the multivariate analysis where a 12% increase in the odds of a MT per 1 year increase in age was observed.

Hormonal exposure is a well-documented canine mammary tumor-associated risk factor and steroid hormones, mainly 17 beta-estradiol (E2), are involved in cell proliferation by exerting an antiapoptotic effect that favors the neoplastic process [10,49]. Furthermore, the landmark publication by Schneider et al., in 1969, reported that mammary tumors occurred in 0.05% of females spayed before the first heat cycle, and this incidence increased from 8% to 26% when the animals were spayed after the first or second heat [16]. As a consequence, reproductive health policies responsible for spaying animals at a very early stage of life had a double beneficial effect, contributing to the reduction in the number of stray dogs and preventing mammary neoplasm development. Likewise, in our study, 83% of mammary neoplasms were diagnosed in unspayed dogs, substantiating the protective effects of ovariohysterectomy as described by several authors [16,18,19].

However, the lack of significance between BTs and MTs and spayed and unspayed dogs could suggest that the hormonal influence sorts an unrelated effect on the CMT malignancy, although 39% of the tumors observed in our cohort of spayed dogs were simple MTs that are generally related to an overall poor prognosis when compared to complex tumors [47,48]. Nevertheless, considering that our dataset lacks information regarding the age of dogs at spaying and that most of the tumors occurred in unspayed dogs probably as a consequence of the ethical concerns in Mediterranean countries regarding the gonadectomy, a careful and prudent outlook should be kept regarding the generalization of the hormonal status role in the onset of CMTs.

The size of the tumor is considered one of the main macroscopical findings related to CMT behavior. In the present study, we also considered the role of the tumor’s size as a clinical, prognosis-related, CMT factor demonstrating that BTs were smaller than MTs, as previously reported by Sonremno et al., 2009 [8]. Furthermore, the tumor’s diameter was related to the histological malignant categories proposed by Pena [24], with small size neoplasm was more prone to a better prognosis compared to the larger one. However, considering the size of the tumor using the WHO classification and the five categories proposed by Sonremno [8], 62.5% of carcinomas were smaller than 3 cm, and 18% were less than 1 cm. Interestingly, these data conflict with what has been described by Sorenmo et al., 2009 [8], who reported that only 3% of MTs were smaller than 1 cm, providing compelling evidence that the tumor size should be carefully evaluated during the assessment of the TNM-WHO clinical staging, as previously suggested by Pena [24].

Supporting these data, the application of the logistic regression characterized the age and the size as the best predictors, with an overall diagnostic accuracy of 0.63 and low predictive values, both positive and negative. This value of accuracy is probably related to the number of factors used in our model. A similar predictive performance was observed using one of the most powerful machine learning models, suggesting that the age and the size are sufficient but not exhaustive parameters for the diagnosis of CMTs. Thanks to dramatic breakthroughs in artificial intelligence and machine learning technologies in the mainstreaming of vertiginous cancer-related research, it is highly credible that the ways to investigate cancer risk factors and the consequently generalized impact will be subverted and revolutionized in a tailor-made personalized animal outlook.

## 5. Conclusions

In conclusion, this multicenter retrospective study, accounting for a large number of CMTs from different academic institutions, offers a unique opportunity to increase the overall knowledge of the main factors involved in canine mammary tumor onset. In our study, the observation that a high number of MTs are smaller than 1 cm suggests the need for a reconsideration of the size (T) parameter in the TNM system and pave the way for the development of tools for the investigation and control of clinical risk factors for small size tumors.

## Figures and Tables

**Table 1:** Model selection.

Model ^1^	Variables ^2^	Likelihood Test ^3^	Wald Test ^4^	Coefficients β_n_^5^	Exp ^6^	Confidence Intervals ^7^
I	Tumor size		8.59; <0.0001	0.24	1.27	1.21–1.35
II	Tumor size	35.48; <0.0001	7.94; <0.0001	0.22	1.25	1.19–1.32
	Age		5.88; <0.0001	0.11	1.12	1.08–1.16
III	Tumor size	0.16; 0.69	7.93; <0.0001	0.22	1.25	1.19–1.32
	Age		5.82; <0.0001	0.11	1.12	1.08–1.16
	Spayed		0.40; 0.69	0.05	1.05	0.82–1.36
IV	Tumor size	0.88; 0.35	7.96; <0.0001	0.22	1.25	1.19–1.32
	Age		5.68; <0.0001	0.11	1.11	1.07–1.16
	Spayed		0.35; 0.73	0.05	1.05	0.81–1.35
	Pure breed		−0.94; 0.35	−0.09	0.91	0.75–1.11

^1^ Models are built so that the smaller models are special cases of the larger ones. Equivalently, the smaller models are obtained by sequentially setting to 0 the coefficients of the full model (IV). The general form is: log-odds = β_0_ + β_1×1_+ β_2_X_2_ + ... + β_n_X_n_: where β_1_, β_2_, ..., β_n_ are the coefficients of the x_1_, x_2_, ..., x_n_ independent variables (covariates) included in the model. odds is calculated according to the formula: odds = exp(β_0_ + β_1_X_1_+ β_2_X_2_ + ... + β_n_X_n_); ^2^ Covariates included in the model. Intercept not reported; ^3^ Likelihood ratio test statistic: Deviance, *p* value; ^4^ Wald test statistic: z and *p* value; ^5^ Model parameters β_n;_^6^ Exponentiated model parameters e βn; ^7^ Wald 95% confidence interval for an exponentiated model parameter.

**Table 2:** Histological diagnosis.

Histological Diagnosis		*N*	%
Simple benign tumors	Adenoma, simple	225	12.1
	Myoepithelioma	3	0.2
Ductal-associated benign tumors	Intraductal papillary adenoma (duct papilloma)	11	0.6
Nonsimple benign tumors	Complex adenoma	281	15.1
	Benign mixed tumor	329	17.6
	Fibroadenoma	18	1.0
Simple carcinoma	Tubular carcinoma	231	12.4
	Tubulopapillary carcinoma	256	13.7
	Solid carcinoma	130	7.0
	Anaplastic carcinoma	7	0.4
Nonsimple carcinoma	Carcinoma arising in a complex adenoma/benign mixed tumor	88	4.7
	Complex carcinoma	248	13.3
	Carcinoma-and-malignant myoepithelioma	5	0.3
Special type	Adenosquamous carcinoma	14	0.8
	Lipidic-rich carcinoma	2	0.1
	Malignant myoepithelioma ^1^	6	0.3
	Mucinous carcinoma	2	0.1
	Spindle cell carcinoma	1	0.1
Others	Osteosarcoma	5	0.3
	Carcinosarcoma	4	0.2
Total		1866	100%

^1^ The diagnosis of malignant myoepithelioma was formulated after performing immunohistochemistry with neoplastic cells positive immunostaining for alpha-smooth actin and P63 and negative stain for luminal epithelial markers.

**Table 3:** Models with categorical covariates.

Model ^1^	Variables ^2^	Wald Test ^3^	Coefficients β_n_^4^	Exp ^5^	Confidence Intervals ^6^
IC	T2	6.152; <0.0001	0.835	2.305	1.77–3.02
	T3	7.086; <0.0001	1.286	3.619	2.56–5.22
	Age 5–8	−0.022; 0.98	−0.005	0.995	0.61–1.62
	Age 9–12	1.872; 0.06	0.456	1.579	0.98–2.56
	Age > 12	3.207; <0.002	0.905	2.472	1.43–4.32
IIC	S2	2.266; <0.03	0.29	1.337	1.04–1.72
	S3	5.069; <0.0001	0.761	2.141	1.60–2.88
	S4	7.257; <0.0001	1.144	3.14	2.31–4.29
	S5	8.036; <0.0001	1.594	4.922	3.36–7.32
	Age 5–8	−0.044; 0.96	−0.011	0.989	0.61–1.62
	Age 9–12	1.751; 0.08	0.431	1.538	0.95–2.50
	Age > 12	2.976; 0.003	0.846	2.331	1.34–4.09

^1.^ Model: Model IC: macroscopic tumor size split according to WHO (T1 < 3 cm, T2 = 3–5 cm, and T3 > 5 cm); Model IIC: macroscopic size split according to Soremno (S1 < 1 cm, S2 = 1 to <2 cm, S3 = 2 to <3 cm, S4 = 3 to < 5 cm, S5 > 5 cm); ^2.^ Covariates included in the model. Reference levels are T1 and age 0–4 years for model IC, tumor size 0–1 cm and age 0–4 years for model IIC; ^3.^ Wald test statistic: z; *P* value; ^4.^ Model parameters β_n_; ^5.^ Exponentiated model parameters e β_n_; ^6.^ Wald 95% confidence interval for an exponentiated model parameter.

**Table 4:** Prediction performances for logistic regression (GLM) and stochastic gradient boosting (GBM). In parentheses 95 percent confidence intervals. Positive class “malignant”.

	AUC (inner) ^1^	AUC ^2^	PPV ^3^	NPV ^4^	Accuracy ^5^
GLM	0.66	0.66	0.65	0.61	0.63
	(0.65–0.67)	(0.63–0.70)	(0.63–0.67)	(0.57–0.64)	(0.60–0.65)
GBM	0.67	0.67	0.65	0.59	0.62
	(0.66–0.67)	(0.64–0.69)	(0.63–0.67)	(0.57–0.62)	(0.61–0.64)

^1^ Average Area Under the receiver operating Characteristic from the inner cross-validation; ^2^ Average Area Under the receiver operating Characteristic from the outer cross-validation; ^3^ Average Positive Predictive Value; ^4^ Average Negative Predictive Value; ^5^ Average Accuracy.

## Supplementary Materials

The following are available online at https://www.mdpi.com/2076-2615/10/9/1687/s1.
