# Supplementary material for: A Statistical Analysis of Risk Factors and Biological Behavior in Canine Mammary Tumors: A Multicenter Study
Source: Animals (Basel). 2020 Sep 18;10(9):1687. doi: 10.3390/ani10091687 (PMC7552647; doi:10.3390/ani10091687)
Supplement: Supplementary file 1 [file animals-10-01687-s001.zip › Logistic Regression supplementary.pdf]

Supplementary Material

Logistic Regression

```
## Model 1
```

```
##
## Call:
## glm(formula = twolevels ~ new_macro_size, family = binomial,
##     data = tr)
##
## Deviance Residuals:
##    Min      1Q  Median      3Q     Max
## -3.713  -1.126   0.723   1.127   1.314
##
## Coefficients:
##                Estimate Std. Error z value Pr(>|z|)
## (Intercept)    -0.36350    0.07236  -5.024 5.07e-07 ***
## new_macro_size  0.24184    0.02815   8.590  < 2e-16 ***
## ---
## Signif. codes:  0 '***' 0.001 '**' 0.01 '*' 0.05 '.' 0.1 ' ' 1
##
## (Dispersion parameter for binomial family taken to be 1)
##
##     Null deviance: 2577.5  on 1865  degrees of freedom
## Residual deviance: 2481.0  on 1864  degrees of freedom
## AIC: 2485
##
## Number of Fisher Scoring iterations: 4
```

```
## Coefficients
```

```
##    (Intercept) new_macro_size
##         -0.364          0.242
```

```
## Exp coefficients
```

```
##    (Intercept) new_macro_size
##          0.695          1.274
```

```
## Exp coefficients confidence intervals
```

```
## Waiting for profiling to be done...
```

```
##                2.5 % 97.5 %
## (Intercept)    0.603  0.800
## new_macro_size 1.207  1.348
```

```
## Model 2
```

```
##
## Call:
## glm(formula = twolevels ~ new_macro_size + age, family = binomial,
##     data = tr)
##
## Deviance Residuals:
##     Min       1Q   Median       3Q      Max
## -3.6877  -1.1271   0.6547   1.0906   1.6819
##
## Coefficients:
##                Estimate Std. Error z value Pr(>|z|)
## (Intercept)    -1.33539    0.18153  -7.356 1.89e-13 ***
## new_macro_size  0.22342    0.02815   7.938 2.05e-15 ***
## age             0.11008    0.01871   5.882 4.06e-09 ***
## ---
## Signif. codes:  0 '***' 0.001 '**' 0.01 '*' 0.05 '.' 0.1 ' ' 1
##
## (Dispersion parameter for binomial family taken to be 1)
##
##     Null deviance: 2577.5  on 1865  degrees of freedom
## Residual deviance: 2445.6  on 1863  degrees of freedom
## AIC: 2451.6
##
## Number of Fisher Scoring iterations: 4
```

```
## Coefficients
```

```
##    (Intercept) new_macro_size            age
##         -1.335          0.223          0.110
```

```
## Exp coefficients
```

```
##    (Intercept) new_macro_size            age
##          0.263          1.250          1.116
```

```
## Exp coefficients confidence intervals
```

```
## Waiting for profiling to be done...
```

```
##                2.5 % 97.5 %
## (Intercept)    0.184  0.375
## new_macro_size 1.185  1.323
## age            1.076  1.158
```

```
## Model 3
```

```
##
## Call:
## glm(formula = twolevels ~ new_macro_size + age + sex, family = binomial,
##     data = tr)
##
## Deviance Residuals:
##     Min       1Q   Median       3Q      Max
## -3.6835  -1.1247   0.6587   1.0943   1.6832
##
## Coefficients:
##                Estimate Std. Error z value Pr(>|z|)
## (Intercept)    -1.33754    0.18162  -7.364 1.78e-13 ***
## new_macro_size  0.22327    0.02815   7.933 2.14e-15 ***
## age             0.10940    0.01879   5.823 5.80e-09 ***
## sexfs           0.05246    0.13008   0.403    0.687
## ---
## Signif. codes:  0 '***' 0.001 '**' 0.01 '*' 0.05 '.' 0.1 ' ' 1
##
## (Dispersion parameter for binomial family taken to be 1)
##
##     Null deviance: 2577.5  on 1865  degrees of freedom
## Residual deviance: 2445.4  on 1862  degrees of freedom
## AIC: 2453.4
##
## Number of Fisher Scoring iterations: 4

## Coefficients

##    (Intercept) new_macro_size            age          sexfs
##         -1.338          0.223          0.109          0.052

## Exp coefficients

##    (Intercept) new_macro_size            age          sexfs
##          0.262          1.250          1.116          1.054

## Exp coefficients confidence intervals

## Waiting for profiling to be done...

##                2.5 % 97.5 %
## (Intercept)    0.183  0.374
## new_macro_size 1.185  1.323
## age            1.075  1.158
## sexfs          0.817  1.361

## Model 4

##
## Call:
## glm(formula = twolevels ~ new_macro_size + age + sex + breed_two,
##     family = binomial, data = tr)
```

```
##
## Deviance Residuals:
##     Min       1Q   Median       3Q      Max
## -3.7033  -1.1280   0.6698   1.1091   1.6923
##
## Coefficients:
##                    Estimate Std. Error z value Pr(>|z|)
## (Intercept)        -1.26254    0.19823  -6.369 1.90e-10 ***
## new_macro_size      0.22408    0.02817   7.956 1.78e-15 ***
## age                 0.10742    0.01891   5.680 1.34e-08 ***
## sexfs               0.04566    0.13030   0.350    0.726
## breed_twopurebreed -0.09343    0.09952  -0.939    0.348
## ---
## Signif. codes:  0 '***' 0.001 '**' 0.01 '*' 0.05 '.' 0.1 ' ' 1
##
## (Dispersion parameter for binomial family taken to be 1)
##
##     Null deviance: 2577.5  on 1865  degrees of freedom
## Residual deviance: 2444.5  on 1861  degrees of freedom
## AIC: 2454.5
##
## Number of Fisher Scoring iterations: 4

## Coefficients

##        (Intercept)     new_macro_size                age              sexfs
##             -1.263              0.224              0.107              0.046
## breed_twopurebreed
##             -0.093

## Exp coefficients

##        (Intercept)     new_macro_size                age              sexfs
##              0.283              1.251              1.113              1.047
## breed_twopurebreed
##              0.911

## Exp coefficients confidence intervals

## Waiting for profiling to be done...

##                    2.5 % 97.5 %
## (Intercept)        0.191  0.416
## new_macro_size     1.186  1.324
## age                1.073  1.156
## sexfs              0.811  1.352
## breed_twopurebreed 0.749  1.107

## Likelihood-Ratio test

## Analysis of Deviance Table
##
```

```
## Model 1: twolevels ~ new_macro_size
## Model 2: twolevels ~ new_macro_size + age
## Model 3: twolevels ~ new_macro_size + age + sex
## Model 4: twolevels ~ new_macro_size + age + sex + breed_two
##   Resid. Df Resid. Dev Df Deviance  Pr(>Chi)
## 1      1864     2481.0
## 2      1863     2445.6  1   35.482 2.574e-09 ***
## 3      1862     2445.4  1    0.163    0.6866
## 4      1861     2444.5  1    0.882    0.3478
## ---
## Signif. codes:  0 '***' 0.001 '**' 0.01 '*' 0.05 '.' 0.1 ' ' 1
```

```
## Analysis of Deviance Table
##
## Model: binomial, link: logit
##
## Response: twolevels
##
## Terms added sequentially (first to last)
##
##
##                Df Deviance Resid. Df Resid. Dev  Pr(>Chi)
## NULL                            1865     2577.5
## new_macro_size  1   96.444      1864     2481.0 < 2.2e-16 ***
## age             1   35.482      1863     2445.6 2.574e-09 ***
## sex             1    0.163      1862     2445.4    0.6866
## breed_two       1    0.882      1861     2444.5    0.3478
## ---
## Signif. codes:  0 '***' 0.001 '**' 0.01 '*' 0.05 '.' 0.1 ' ' 1
```

Categorized

```
## Model IC
```

```
##
## Call:
## glm(formula = twolevels ~ TNM + age_groups, family = binomial,
##     data = tr)
##
## Deviance Residuals:
##     Min       1Q   Median       3Q      Max
## -1.9607  -1.1900   0.6894   1.1649   1.3657
##
## Coefficients:
##                  Estimate Std. Error z value Pr(>|z|)
## (Intercept)     -0.426956   0.235037  -1.817  0.06929 .
## TNMT2            0.835155   0.135762   6.152 7.67e-10 ***
## TNMT3            1.286219   0.181528   7.086 1.39e-12 ***
## age_groups5-8   -0.005382   0.247179  -0.022  0.98263
## age_groups9-12   0.456486   0.243882   1.872  0.06124 .
## age_groups13-22  0.904838   0.282107   3.207  0.00134 **
## ---
```

```
## Signif. codes:  0 '***' 0.001 '**' 0.01 '*' 0.05 '.' 0.1 ' ' 1
##
## (Dispersion parameter for binomial family taken to be 1)
##
##     Null deviance: 2577.5  on 1865  degrees of freedom
## Residual deviance: 2439.1  on 1860  degrees of freedom
## AIC: 2451.1
##
## Number of Fisher Scoring iterations: 4
```

```
## Coefficients
```

```
##     (Intercept)           TNMT2           TNMT3   age_groups5-8  age_groups9-12
##          -0.427           0.835           1.286          -0.005           0.456
## age_groups13-22
##           0.905
```

```
## Exp coefficients
```

```
##     (Intercept)           TNMT2           TNMT3   age_groups5-8  age_groups9-12
##           0.652           2.305           3.619           0.995           1.579
## age_groups13-22
##           2.472
```

```
## Exp coefficients confidence intervals
```

```
## Waiting for profiling to be done...
```

```
##                 2.5 % 97.5 %
## (Intercept)     0.409  1.031
## TNMT2           1.771  3.017
## TNMT3           2.557  5.217
## age_groups5-8   0.614  1.623
## age_groups9-12  0.981  2.560
## age_groups13-22 1.426  4.320
```

```
## Model IIC
```

```
##
## Call:
## glm(formula = twolevels ~ srn + age_groups, family = binomial,
##     data = tr)
##
## Deviance Residuals:
##     Min       1Q   Median       3Q      Max
## -1.9447  -1.0603   0.6906   1.1213   1.4960
##
## Coefficients:
##                 Estimate Std. Error z value Pr(>|z|)
## (Intercept)      -0.7126     0.2494  -2.858  0.00426 **
## srn1-2            0.2901     0.1280   2.266  0.02346 *
## srn2-3            0.7612     0.1502   5.069 4.01e-07 ***
```

```
## srn3-5            1.1441     0.1577   7.257 3.97e-13 ***
## srn>5             1.5938     0.1983   8.036 9.29e-16 ***
## age_groups5-8    -0.0110     0.2492  -0.044  0.96479
## age_groups9-12    0.4307     0.2459   1.751  0.07989 .
## age_groups13-22   0.8462     0.2843   2.976  0.00292 **
## ---
## Signif. codes:  0 '***' 0.001 '**' 0.01 '*' 0.05 '.' 0.1 ' ' 1
##
## (Dispersion parameter for binomial family taken to be 1)
##
##     Null deviance: 2577.5  on 1865  degrees of freedom
## Residual deviance: 2413.0  on 1858  degrees of freedom
## AIC: 2429
##
## Number of Fisher Scoring iterations: 4
```

```
## Coefficients
```

```
##     (Intercept)          srn1-2          srn2-3          srn3-5           srn>5
##          -0.713           0.290           0.761           1.144           1.594
##   age_groups5-8  age_groups9-12 age_groups13-22
##          -0.011           0.431           0.846
```

```
## Exp coefficients
```

```
##     (Intercept)          srn1-2          srn2-3          srn3-5           srn>5
##           0.490           1.337           2.141           3.140           4.922
##   age_groups5-8  age_groups9-12 age_groups13-22
##           0.989           1.538           2.331
```

```
## Exp coefficients confidence intervals
```

```
## Waiting for profiling to be done...
```

```
##                 2.5 % 97.5 %
## (Intercept)     0.299  0.797
## srn1-2          1.040  1.719
## srn2-3          1.597  2.878
## srn3-5          2.310  4.288
## srn>5           3.361  7.324
## age_groups5-8   0.608  1.620
## age_groups9-12  0.952  2.504
## age_groups13-22 1.339  4.091
```

```
## R version 3.6.3 (2020-02-29)
## Platform: x86_64-w64-mingw32/x64 (64-bit)
## Running under: Windows 10 x64 (build 19041)
##
## Matrix products: default
##
## locale:
## [1] LC_COLLATE=Italian_Italy.1252  LC_CTYPE=Italian_Italy.1252
```

```
## [3] LC_MONETARY=Italian_Italy.1252 LC_NUMERIC=C
## [5] LC_TIME=Italian_Italy.1252
##
## attached base packages:
## [1] stats     graphics  grDevices utils     datasets  methods   base
##
## other attached packages:
## [1] pacman_0.5.1
##
## loaded via a namespace (and not attached):
##  [1] MASS_7.3-51.5   compiler_3.6.3  magrittr_1.5    tools_3.6.3
##  [5] htmltools_0.4.0 yaml_2.2.1      Rcpp_1.0.3      stringi_1.4.6
##  [9] rmarkdown_2.1   knitr_1.28      stringr_1.4.0   xfun_0.12
## [13] digest_0.6.25   rlang_0.4.5     evaluate_0.14
```
