# Supplementary material for: A Statistical Analysis of Risk Factors and Biological Behavior in Canine Mammary Tumors: A Multicenter Study
Source: Animals (Basel). 2020 Sep 18;10(9):1687. doi: 10.3390/ani10091687 (PMC7552647; doi:10.3390/ani10091687)
Supplement: Supplementary file 1 [file animals-10-01687-s001.zip › Machine Learning model supplementary.pdf]

Supplementary Material

Import required libraries

```
if (!require("pacman")) install.packages("pacman")
```

```
## Loading required package: pacman
```

```
pacman::p_load(caret)
pacman::p_load(pROC)
pacman::p_load(ggplot2)
pacman::p_load(dplyr)


print(getwd())
```

Inputs

```
rSeed <- 42
rTuneLength <- 10
outerFolds <- 5
savePrefix <- "00"
factors <- c( 'new_macro_size', 'age', 'factor(sex)', "factor(breed_two)")
selected_names <- c( 'twolevels', 'new_macro_size', 'age', 'sex', "breed_two")
strFormula <- paste(paste("twolevels", "~"), paste(factors, collapse="+"))
formulaFit <- as.formula(strFormula)
formulaFit
pos_class <- 'malignant'
outfname <- paste0(savePrefix, ".out")
write(outfname, outfname, append = FALSE)
```

```
ltr <- read.csv("../database_clean.csv")
cat('Total ', nrow(ltr))
```

```
## Total  1866
```

```
#print(colnames(ltr))

set.seed(rSeed)
inTrain <- createFolds(y=ltr$twolevels, k=outerFolds, list=TRUE, returnTrain = TRUE)
```

```r
for(fold in inTrain) {
  training <- ltr[fold, ]
  cat('\nTraining ', nrow(training))
  test     <- ltr[-fold, ]
  cat('\nTest ', nrow(test))
}
```

```
##
## Training  1492
## Test  374
## Training  1494
## Test  372
## Training  1493
## Test  373
## Training  1493
## Test  373
## Training  1492
## Test  374
```

Setup inner 10-fold cross validation repeated 5 times

```r
fitControl <- trainControl(## 10 -fold CV
                           method = "repeatedcv",
                           number = 10,
                           ## repeated five times
                           repeats = 5,
                           ## request class probabilities, required for using ROC
                           classProbs = TRUE,
                           returnResamp = 'final',
                           savePredictions = 'final',
                           summaryFunction = twoClassSummary)
```

```r
getResults <- function(confusionMatrices) {
  print("Total")
  overallCm <- Reduce('+', lapply(confusionMatrices, function(x) return(x$table)))
  cm <- confusionMatrix(overallCm, positive = pos_class)
  print(cm)
  print("Average")
  overallCm <- Reduce('+', lapply(confusionMatrices, function(x) return(x$table)))
  cm <- confusionMatrix(round(overallCm/length(inTrain), digits = 0), positive = pos_class)
  print(cm)
  print("Single averages")
  ll <- lapply(confusionMatrices, function(x) return(x$overall))
  overall <- data.frame(matrix(unlist(ll), nrow=length(ll), byrow = T))
  colnames(overall) <- names(ll[[1]])
  print(t.test(overall$Accuracy))
  ll <- lapply(confusionMatrices, function(x) return(x$byClass))
  overall <- data.frame(matrix(unlist(ll), nrow=length(ll), byrow = T))
  colnames(overall) <- names(ll[[1]])
  print(t.test(overall$`Balanced Accuracy`))
```

```
  print(t.test(overall$`Pos Pred Value`))
  print(t.test(overall$`Neg Pred Value`))
}
```

GLM

```
confusionMatricesGlm <- list()
confusionMatricesGlmTrain <- list()

fullGlmFit <- list()
aucsTrain <- list()
aucs <- list()

i <- 1
for (fold in inTrain) {
  training <- ltr[fold, selected_names]
  test     <- ltr[-fold, selected_names]
  set.seed(rSeed)
  foldGlmFit <- train(formulaFit, data = training,
              method="glm", family=binomial,
              preProc = c("center", "scale"),
              trControl = fitControl,
              metric = "ROC")
  fullGlmFit[[i]] <- foldGlmFit
  sprintf(fmt = "Fold  %d\n", i) %>% cat()
  sprintf(fmt = "Training summary\n") %>% cat()
  print(summary(foldGlmFit))
  cmt <- confusionMatrix.train(foldGlmFit)
  cmt <-confusionMatrix(cmt$table, positive = pos_class)
  confusionMatricesGlmTrain[[i]] <- cmt
  print(cmt)
  aucsTrain[[i]] <-foldGlmFit$results$ROC
  sprintf(fmt = "AUC inner %f\n", foldGlmFit$results$ROC) %>% cat()

  #saveRDS(foldGlmFit, file=paste0(savePrefix, "foldGlm.rds"))
  sprintf(fmt = "\nTesting summary\n") %>% cat()
  predictions <- predict(foldGlmFit, newdata=select(test,-c(twolevels)))
  cm <- confusionMatrix(predictions, test$twolevels, positive = pos_class)
  confusionMatricesGlm[[i]] <- cm

  prob_predictions <- predict(foldGlmFit, newdata=select(test,-c(twolevels)), type="prob")[, pos_class]
  objROC <- roc(test$twolevels, prob_predictions, auc=TRUE, ci=TRUE)
  print(objROC)
  aucs[[i]] <- objROC$auc

  i <- i + 1
  print(cm)
  sprintf(fmt = "=================================================================\n") %>% cat()
}
```

```
## Fold  1
```

```
## Training summary
##
## Call:
## NULL
##
## Deviance Residuals:
##     Min       1Q   Median       3Q      Max
## -3.5930  -1.1321   0.6562   1.0986   1.6178
##
## Coefficients:
##                             Estimate Std. Error z value Pr(>|z|)
## (Intercept)                  0.17808    0.05456   3.264   0.0011 **
## new_macro_size               0.52387    0.07614   6.880 5.97e-12 ***
## age                          0.31620    0.05593   5.653 1.57e-08 ***
## `factor(sex)fs`              0.03954    0.05449   0.726   0.4680
## `factor(breed_two)purebreed` -0.01720    0.05436  -0.316   0.7517
## ---
## Signif. codes:  0 '***' 0.001 '**' 0.01 '*' 0.05 '.' 0.1 ' ' 1
##
## (Dispersion parameter for binomial family taken to be 1)
##
##     Null deviance: 2060.8  on 1491  degrees of freedom
## Residual deviance: 1950.3  on 1487  degrees of freedom
## AIC: 1960.3
##
## Number of Fisher Scoring iterations: 4
##
## Confusion Matrix and Statistics
##
##            Reference
## Prediction    benign malignant
##   benign    26.74263  18.20375
##   malignant 19.70509  35.34853
##
##                Accuracy : 0.6209
##                  95% CI : (NA, NA)
##     No Information Rate : NA
##     P-Value [Acc > NIR] : NA
##
##                   Kappa : 0.2363
##
##  Mcnemar's Test P-Value : 0.9351
##
##             Sensitivity : 0.6601
##             Specificity : 0.5758
##          Pos Pred Value : 0.6421
##          Neg Pred Value : 0.5950
##              Prevalence : 0.5355
##          Detection Rate : 0.3535
##    Detection Prevalence : 0.5505
##       Balanced Accuracy : 0.6179
##
##        'Positive' Class : malignant
##
```

```
## AUC inner 0.662682
##
## Testing summary

## Setting levels: control = benign, case = malignant

## Setting direction: controls < cases

##
## Call:
## roc.default(response = test$twolevels, predictor = prob_predictions,     auc = TRUE, ci = TRUE)
##
## Data: prob_predictions in 174 controls (test$twolevels benign) < 200 cases (test$twolevels malignant
## Area under the curve: 0.6408
## 95% CI: 0.585-0.6966 (DeLong)
## Confusion Matrix and Statistics
##
##            Reference
## Prediction  benign malignant
##   benign        94        66
##   malignant     80       134
##
##                Accuracy : 0.6096
##                  95% CI : (0.5581, 0.6594)
##     No Information Rate : 0.5348
##     P-Value [Acc > NIR] : 0.00209
##
##                   Kappa : 0.2113
##
##  Mcnemar's Test P-Value : 0.28198
##
##             Sensitivity : 0.6700
##             Specificity : 0.5402
##          Pos Pred Value : 0.6262
##          Neg Pred Value : 0.5875
##              Prevalence : 0.5348
##          Detection Rate : 0.3583
##    Detection Prevalence : 0.5722
##       Balanced Accuracy : 0.6051
##
##        'Positive' Class : malignant
##
## =================================================================
## Fold  2
## Training summary
##
## Call:
## NULL
##
## Deviance Residuals:
##     Min       1Q   Median       3Q      Max
## -3.5390  -1.1499   0.6942   1.1180   1.6687
##
```

```
## Coefficients:
##                               Estimate Std. Error z value Pr(>|z|)
## (Intercept)                    0.16973    0.05404   3.141  0.00168 **
## new_macro_size                 0.46920    0.07072   6.635 3.24e-11 ***
## age                            0.26788    0.05547   4.830 1.37e-06 ***
## `factor(sex)fs`                0.03199    0.05396   0.593  0.55329
## `factor(breed_two)purebreed` -0.07084    0.05409  -1.310  0.19030
## ---
## Signif. codes:  0 '***' 0.001 '**' 0.01 '*' 0.05 '.' 0.1 ' ' 1
##
## (Dispersion parameter for binomial family taken to be 1)
##
##     Null deviance: 2063.6  on 1493  degrees of freedom
## Residual deviance: 1968.6  on 1489  degrees of freedom
## AIC: 1978.6
##
## Number of Fisher Scoring iterations: 4
##
## Confusion Matrix and Statistics
##
##            Reference
## Prediction    benign malignant
##   benign    25.63588  17.69746
##   malignant 20.81660  35.85007
##
##                Accuracy : 0.6149
##                  95% CI : (NA, NA)
##     No Information Rate : NA
##     P-Value [Acc > NIR] : NA
##
##                   Kappa : 0.2224
##
##  Mcnemar's Test P-Value : 0.7328
##
##             Sensitivity : 0.6695
##             Specificity : 0.5519
##          Pos Pred Value : 0.6326
##          Neg Pred Value : 0.5916
##              Prevalence : 0.5355
##          Detection Rate : 0.3585
##    Detection Prevalence : 0.5667
##       Balanced Accuracy : 0.6107
##
##        'Positive' Class : malignant
##
## AUC inner 0.650998
##
## Testing summary

## Setting levels: control = benign, case = malignant
## Setting direction: controls < cases

##
## Call:
```

```
## roc.default(response = test$twolevels, predictor = prob_predictions,     auc = TRUE, ci = TRUE)
##
## Data: prob_predictions in 173 controls (test$twolevels benign) < 199 cases (test$twolevels malignant)
## Area under the curve: 0.6955
## 95% CI: 0.6417-0.7493 (DeLong)
## Confusion Matrix and Statistics
##
##            Reference
## Prediction  benign malignant
##   benign       102        55
##   malignant     71       144
##
##                Accuracy : 0.6613
##                  95% CI : (0.6107, 0.7093)
##     No Information Rate : 0.5349
##     P-Value [Acc > NIR] : 5.122e-07
##
##                   Kappa : 0.3151
##
##  Mcnemar's Test P-Value : 0.1814
##
##             Sensitivity : 0.7236
##             Specificity : 0.5896
##          Pos Pred Value : 0.6698
##          Neg Pred Value : 0.6497
##              Prevalence : 0.5349
##          Detection Rate : 0.3871
##    Detection Prevalence : 0.5780
##       Balanced Accuracy : 0.6566
##
##        'Positive' Class : malignant
##
## ==================================================================
## Fold  3
## Training summary
##
## Call:
## NULL
##
## Deviance Residuals:
##     Min       1Q   Median       3Q      Max
## -3.8669  -1.1242   0.6539   1.1020   1.7215
##
## Coefficients:
##                              Estimate Std. Error z value Pr(>|z|)
## (Intercept)                   0.18001    0.05467   3.293 0.000992 ***
## new_macro_size                0.57456    0.07740   7.424 1.14e-13 ***
## age                           0.29583    0.05593   5.290 1.23e-07 ***
## `factor(sex)fs`               0.01019    0.05450   0.187 0.851692
## `factor(breed_two)purebreed` -0.04701    0.05447  -0.863 0.388060
## ---
## Signif. codes:  0 '***' 0.001 '**' 0.01 '*' 0.05 '.' 0.1 ' ' 1
##
## (Dispersion parameter for binomial family taken to be 1)
```

```
##
##     Null deviance: 2062.3  on 1492  degrees of freedom
## Residual deviance: 1947.8  on 1488  degrees of freedom
## AIC: 1957.8
##
## Number of Fisher Scoring iterations: 4
##
## Confusion Matrix and Statistics
##
##            Reference
## Prediction    benign malignant
##   benign    27.20697  17.81648
##   malignant 19.27662  35.69993
##
##                Accuracy : 0.6291
##                  95% CI : (NA, NA)
##     No Information Rate : NA
##     P-Value [Acc > NIR] : NA
##
##                   Kappa : 0.2529
##
##  Mcnemar's Test P-Value : 0.9398
##
##             Sensitivity : 0.6671
##             Specificity : 0.5853
##          Pos Pred Value : 0.6494
##          Neg Pred Value : 0.6043
##              Prevalence : 0.5352
##          Detection Rate : 0.3570
##    Detection Prevalence : 0.5498
##       Balanced Accuracy : 0.6262
##
##        'Positive' Class : malignant
##
## AUC inner 0.669856
##
## Testing summary

## Setting levels: control = benign, case = malignant
## Setting direction: controls < cases

##
## Call:
## roc.default(response = test$twolevels, predictor = prob_predictions,     auc = TRUE, ci = TRUE)
##
## Data: prob_predictions in 173 controls (test$twolevels benign) < 200 cases (test$twolevels malignant
## Area under the curve: 0.6465
## 95% CI: 0.5905-0.7025 (DeLong)
## Confusion Matrix and Statistics
##
##            Reference
## Prediction  benign malignant
##   benign       108        77
##   malignant     65       123
```

```
##
##                Accuracy : 0.6193
##                  95% CI : (0.5679, 0.6688)
##     No Information Rate : 0.5362
##     P-Value [Acc > NIR] : 0.0007242
##
##                   Kappa : 0.2382
##
##  Mcnemar's Test P-Value : 0.3559554
##
##             Sensitivity : 0.6150
##             Specificity : 0.6243
##          Pos Pred Value : 0.6543
##          Neg Pred Value : 0.5838
##              Prevalence : 0.5362
##          Detection Rate : 0.3298
##    Detection Prevalence : 0.5040
##       Balanced Accuracy : 0.6196
##
##        'Positive' Class : malignant
##
## ================================================================
## Fold  4
## Training summary
##
## Call:
## NULL
##
## Deviance Residuals:
##     Min       1Q   Median       3Q      Max
## -3.5920  -1.1343   0.6943   1.1130   1.6217
##
## Coefficients:
##                              Estimate Std. Error z value Pr(>|z|)
## (Intercept)                   0.17484    0.05425   3.223  0.00127 **
## new_macro_size                0.52657    0.07614   6.916 4.64e-12 ***
## age                           0.24736    0.05524   4.478 7.53e-06 ***
## `factor(sex)fs`              -0.03493    0.05387  -0.648  0.51667
## `factor(breed_two)purebreed` -0.04845    0.05411  -0.895  0.37057
## ---
## Signif. codes:  0 '***' 0.001 '**' 0.01 '*' 0.05 '.' 0.1 ' ' 1
##
## (Dispersion parameter for binomial family taken to be 1)
##
##     Null deviance: 2062.3  on 1492  degrees of freedom
## Residual deviance: 1967.2  on 1488  degrees of freedom
## AIC: 1977.2
##
## Number of Fisher Scoring iterations: 4
##
## Confusion Matrix and Statistics
##
##            Reference
## Prediction    benign malignant
```

```
##   benign    26.72472  17.96383
##   malignant 19.75887  35.55258
##
##                Accuracy : 0.6228
##                  95% CI : (NA, NA)
##     No Information Rate : NA
##     P-Value [Acc > NIR] : NA
##
##                   Kappa : 0.2399
##
##  Mcnemar's Test P-Value : 0.897
##
##             Sensitivity : 0.6643
##             Specificity : 0.5749
##          Pos Pred Value : 0.6428
##          Neg Pred Value : 0.5980
##              Prevalence : 0.5352
##          Detection Rate : 0.3555
##    Detection Prevalence : 0.5531
##       Balanced Accuracy : 0.6196
##
##        'Positive' Class : malignant
##
## AUC inner 0.654096
##
## Testing summary

## Setting levels: control = benign, case = malignant
## Setting direction: controls < cases

##
## Call:
## roc.default(response = test$twolevels, predictor = prob_predictions,     auc = TRUE, ci = TRUE)
##
## Data: prob_predictions in 173 controls (test$twolevels benign) < 200 cases (test$twolevels malignant)
## Area under the curve: 0.6926
## 95% CI: 0.6394-0.7458 (DeLong)
## Confusion Matrix and Statistics
##
##            Reference
## Prediction  benign malignant
##   benign       102        65
##   malignant     71       135
##
##                Accuracy : 0.6354
##                  95% CI : (0.5843, 0.6843)
##     No Information Rate : 0.5362
##     P-Value [Acc > NIR] : 6.713e-05
##
##                   Kappa : 0.2652
##
##  Mcnemar's Test P-Value : 0.6681
##
##             Sensitivity : 0.6750
```

```
##             Specificity : 0.5896
##          Pos Pred Value : 0.6553
##          Neg Pred Value : 0.6108
##              Prevalence : 0.5362
##          Detection Rate : 0.3619
##    Detection Prevalence : 0.5523
##       Balanced Accuracy : 0.6323
##
##        'Positive' Class : malignant
##
## ================================================================
## Fold  5
## Training summary
##
## Call:
## NULL
##
## Deviance Residuals:
##     Min       1Q   Median       3Q      Max
## -2.7006  -1.1203   0.6431   1.1089   1.7279
##
## Coefficients:
##                              Estimate Std. Error z value Pr(>|z|)
## (Intercept)                   0.18402    0.05488   3.353 0.000799 ***
## new_macro_size                0.59066    0.07593   7.779 7.31e-15 ***
## age                           0.28817    0.05584   5.160 2.46e-07 ***
## `factor(sex)fs`               0.04054    0.05468   0.741 0.458453
## `factor(breed_two)purebreed` -0.04617    0.05439  -0.849 0.395952
## ---
## Signif. codes:  0 '***' 0.001 '**' 0.01 '*' 0.05 '.' 0.1 ' ' 1
##
## (Dispersion parameter for binomial family taken to be 1)
##
##     Null deviance: 2060.8  on 1491  degrees of freedom
## Residual deviance: 1938.8  on 1487  degrees of freedom
## AIC: 1948.8
##
## Number of Fisher Scoring iterations: 4
##
## Confusion Matrix and Statistics
##
##            Reference
## Prediction    benign malignant
##   benign    27.96247  18.60590
##   malignant 18.48525  34.94638
##
##                Accuracy : 0.6291
##                  95% CI : (NA, NA)
##     No Information Rate : NA
##     P-Value [Acc > NIR] : NA
##
##                   Kappa : 0.2545
##
##  Mcnemar's Test P-Value : 0.8852
```

```
##
##             Sensitivity : 0.6526
##             Specificity : 0.6020
##          Pos Pred Value : 0.6540
##          Neg Pred Value : 0.6005
##              Prevalence : 0.5355
##          Detection Rate : 0.3495
##    Detection Prevalence : 0.5343
##       Balanced Accuracy : 0.6273
##
##        'Positive' Class : malignant
##
## AUC inner 0.668021
##
## Testing summary
```

```
## Setting levels: control = benign, case = malignant
## Setting direction: controls < cases
```

```
##
## Call:
## roc.default(response = test$twolevels, predictor = prob_predictions,     auc = TRUE, ci = TRUE)
##
## Data: prob_predictions in 174 controls (test$twolevels benign) < 200 cases (test$twolevels malignant
## Area under the curve: 0.6465
## 95% CI: 0.5907-0.7022 (DeLong)
## Confusion Matrix and Statistics
##
##            Reference
## Prediction  benign malignant
##   benign        97        64
##   malignant     77       136
##
##                Accuracy : 0.623
##                  95% CI : (0.5717, 0.6723)
##     No Information Rate : 0.5348
##     P-Value [Acc > NIR] : 0.0003496
##
##                   Kappa : 0.2386
##
##  Mcnemar's Test P-Value : 0.3122164
##
##             Sensitivity : 0.6800
##             Specificity : 0.5575
##          Pos Pred Value : 0.6385
##          Neg Pred Value : 0.6025
##              Prevalence : 0.5348
##          Detection Rate : 0.3636
##    Detection Prevalence : 0.5695
##       Balanced Accuracy : 0.6187
##
##        'Positive' Class : malignant
##
## ================================================================
```

```
## [1] "Total"
## Confusion Matrix and Statistics
##
##            Reference
## Prediction  benign malignant
##   benign       503       327
##   malignant    364       672
##
##                Accuracy : 0.6297
##                  95% CI : (0.6073, 0.6516)
##     No Information Rate : 0.5354
##     P-Value [Acc > NIR] : <2e-16
##
##                   Kappa : 0.2535
##
##  Mcnemar's Test P-Value : 0.1708
##
##             Sensitivity : 0.6727
##             Specificity : 0.5802
##          Pos Pred Value : 0.6486
##          Neg Pred Value : 0.6060
##              Prevalence : 0.5354
##          Detection Rate : 0.3601
##    Detection Prevalence : 0.5552
##       Balanced Accuracy : 0.6264
##
##        'Positive' Class : malignant
##
## [1] "Average"
## Confusion Matrix and Statistics
##
##            Reference
## Prediction  benign malignant
##   benign       101        65
##   malignant     73       134
##
##                Accuracy : 0.63
##                  95% CI : (0.5788, 0.6792)
##     No Information Rate : 0.5335
##     P-Value [Acc > NIR] : 0.0001036
##
##                   Kappa : 0.2546
##
##  Mcnemar's Test P-Value : 0.5512557
##
##             Sensitivity : 0.6734
##             Specificity : 0.5805
##          Pos Pred Value : 0.6473
##          Neg Pred Value : 0.6084
##              Prevalence : 0.5335
##          Detection Rate : 0.3592
##    Detection Prevalence : 0.5550
##       Balanced Accuracy : 0.6269
##
```

```
##        'Positive' Class : malignant
##
## [1] "Single averages"
##
##  One Sample t-test
##
## data:  overall$Accuracy
## t = 70.707, df = 4, p-value = 2.397e-07
## alternative hypothesis: true mean is not equal to 0
## 95 percent confidence interval:
##  0.6049934 0.6544476
## sample estimates:
## mean of x
## 0.6297205
##
##
##  One Sample t-test
##
## data:  overall$`Balanced Accuracy`
## t = 72.226, df = 4, p-value = 2.202e-07
## alternative hypothesis: true mean is not equal to 0
## 95 percent confidence interval:
##  0.6023963 0.6505612
## sample estimates:
## mean of x
## 0.6264787
##
##
##  One Sample t-test
##
## data:  overall$`Pos Pred Value`
## t = 86.297, df = 4, p-value = 1.081e-07
## alternative hypothesis: true mean is not equal to 0
## 95 percent confidence interval:
##  0.6279315 0.6696799
## sample estimates:
## mean of x
## 0.6488057
##
##
##  One Sample t-test
##
## data:  overall$`Neg Pred Value`
## t = 51.514, df = 4, p-value = 8.499e-07
## alternative hypothesis: true mean is not equal to 0
## 95 percent confidence interval:
##  0.5741383 0.6395530
## sample estimates:
## mean of x
## 0.6068456
```

```
## [1] "Mean AUCs train"
```

```
##
```

```
##  One Sample t-test
##
## data:  unlist(aucsTrain)
## t = 177.27, df = 4, p-value = 6.074e-09
## alternative hypothesis: true mean is not equal to 0
## 95 percent confidence interval:
##  0.6507762 0.6714855
## sample estimates:
## mean of x
## 0.6611308

## [1] "Mean AUCs test"

##
##  One Sample t-test
##
## data:  unlist(aucs)
## t = 54.614, df = 4, p-value = 6.729e-07
## alternative hypothesis: true mean is not equal to 0
## 95 percent confidence interval:
##  0.6305994 0.6981504
## sample estimates:
## mean of x
## 0.6643749
```

Gradient boosting

```
gbmGrid <-  expand.grid(interaction.depth = c(1, 5, 10, 15),
                    n.trees = (1:25)*200,
                    shrinkage = c(.01, .1, .2),
                    n.minobsinnode = c(5, 10, 15))

gbmGridBest <- expand.grid(interaction.depth = c(1),
                    n.trees = c(400, 600, 800),
                    shrinkage = c(.01),
                    n.minobsinnode = c(5, 10, 15, 20))



confusionMatricesGbm <- list()
confusionMatricesGbmTrain <- list()
fullGbmFit <- list()
gbmaucsTrain <- list()
gbmaucs <- list()
gbmsm <- list()

i <- 1
for (fold in inTrain) {
  training <- ltr[fold, selected_names]
  test     <- ltr[-fold, selected_names]
  set.seed(rSeed)
```

```r
  foldGbmFit <- train(formulaFit, data=training,
                      method='gbm',
                      preProc = c("center", "scale"),
                      trControl = fitControl,
                      metric='ROC',
                      verbose=FALSE,
                      #tuneLength = 2)
                      tuneGrid = gbmGridBest)
                      #tuneGrid = gbmGrid)
                      #tuneLength = rTuneLength)

  fullGbmFit[[i]] <- foldGbmFit
  sprintf(fmt = "Fold  %d\n", i) %>% cat()
  sprintf(fmt = "Training summary\n") %>% cat()
  #print(summary(foldGbmFit))
  gbmsm[[i]] <- summary(foldGbmFit)
  #print(foldGbmFit)
  print(plot(foldGbmFit))#, metric='ROC')

  cmt <- confusionMatrix.train(foldGbmFit)
  cmt <-confusionMatrix(cmt$table, positive = pos_class)
  confusionMatricesGbmTrain[[i]] <- cmt
  print(cmt)
  gbmaucsTrain[[i]] <-max(foldGbmFit$results$ROC)
  sprintf(fmt = "AUC inner %f\n", max(foldGbmFit$results$ROC)) %>% cat()


  sprintf(fmt = "\nTesting summary\n") %>% cat()
  predictions <- predict(foldGbmFit, newdata=select(test,-c(twolevels)))
  cm <- confusionMatrix(predictions, test$twolevels, positive = pos_class)
  confusionMatricesGbm[[i]] <- cm

  prob_predictions <- predict(foldGbmFit, newdata=select(test,-c(twolevels)), type="prob")[, pos_class]
  objROC <- roc(test$twolevels, prob_predictions, auc=TRUE, ci=TRUE)
  print(objROC)
  gbmaucs[[i]] <- objROC$auc

  i <- i + 1
  print(cm)
  sprintf(fmt = "=================================================================\n") %>% cat()
}
```

```
## Fold  1
## Training summary
```

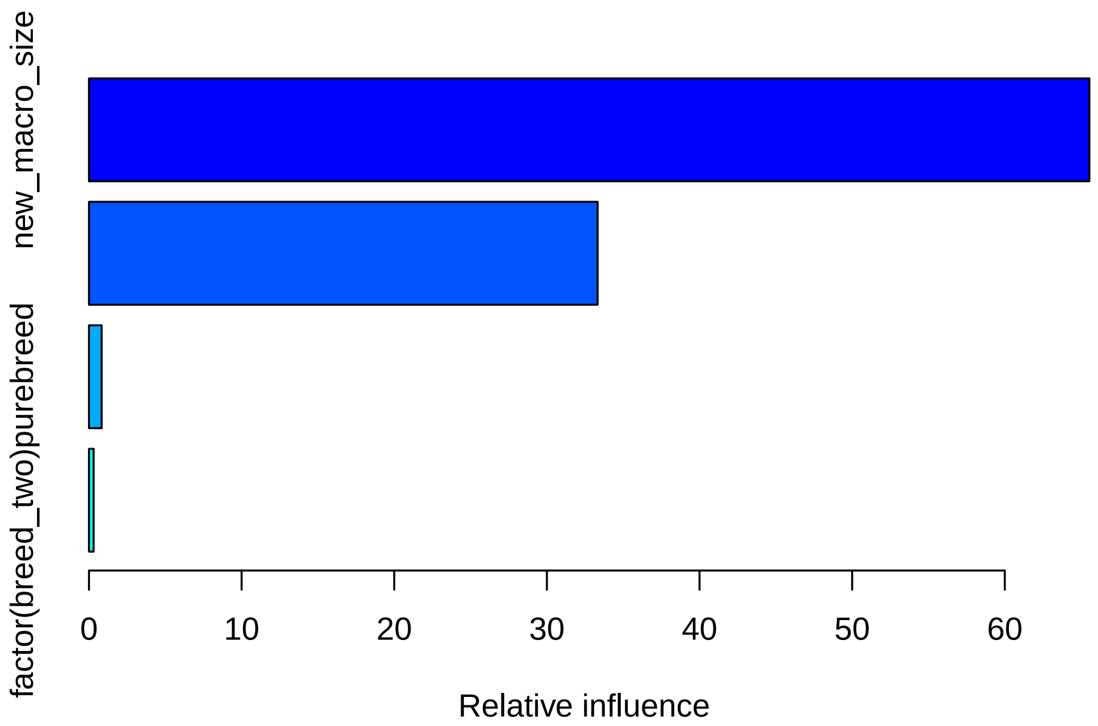


```
## Confusion Matrix and Statistics
##
##            Reference
## Prediction    benign malignant
##   benign    27.84182  18.33780
##   malignant 18.60590  35.21448
##
##                Accuracy : 0.6306
##                  95% CI : (NA, NA)
##     No Information Rate : NA
##     P-Value [Acc > NIR] : NA
##
##                   Kappa : 0.2571
##
##  Mcnemar's Test P-Value : 0.9042
##
##             Sensitivity : 0.6576
##             Specificity : 0.5994
##          Pos Pred Value : 0.6543
##          Neg Pred Value : 0.6029
##              Prevalence : 0.5355
##          Detection Rate : 0.3521
##    Detection Prevalence : 0.5382
##       Balanced Accuracy : 0.6285
##
##        'Positive' Class : malignant
```

```
##
## AUC inner 0.671322
##
## Testing summary

## Setting levels: control = benign, case = malignant

## Setting direction: controls < cases
```

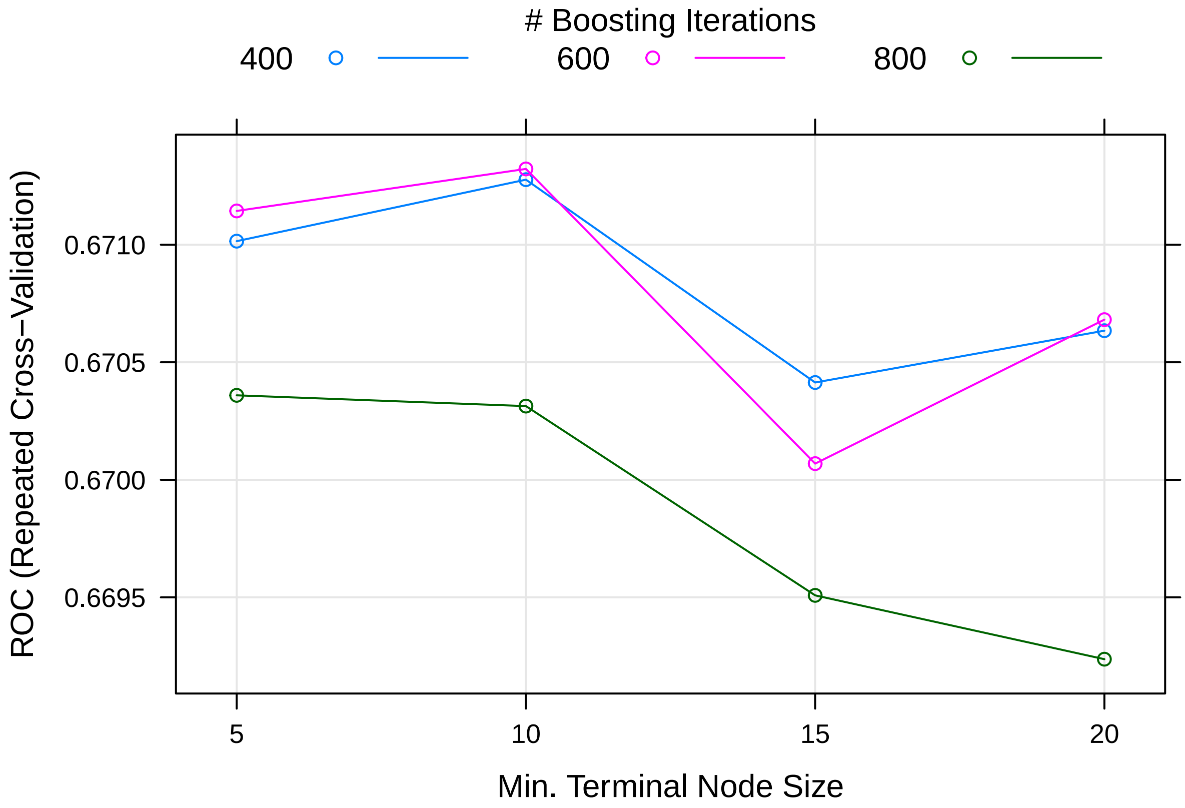


```
##
## Call:
## roc.default(response = test$twolevels, predictor = prob_predictions,     auc = TRUE, ci = TRUE)
##
## Data: prob_predictions in 174 controls (test$twolevels benign) < 200 cases (test$twolevels malignant)
## Area under the curve: 0.6436
## 95% CI: 0.5879-0.6994 (DeLong)
## Confusion Matrix and Statistics
##
##            Reference
## Prediction  benign malignant
##   benign       102        78
##   malignant     72       122
##
##                Accuracy : 0.5989
##                  95% CI : (0.5473, 0.649)
```

```
##     No Information Rate : 0.5348
##     P-Value [Acc > NIR] : 0.007244
##
##                   Kappa : 0.1958
##
##  Mcnemar's Test P-Value : 0.683091
##
##             Sensitivity : 0.6100
##             Specificity : 0.5862
##          Pos Pred Value : 0.6289
##          Neg Pred Value : 0.5667
##              Prevalence : 0.5348
##          Detection Rate : 0.3262
##    Detection Prevalence : 0.5187
##       Balanced Accuracy : 0.5981
##
##        'Positive' Class : malignant
##
## =================================================================
## Fold  2
## Training summary
```

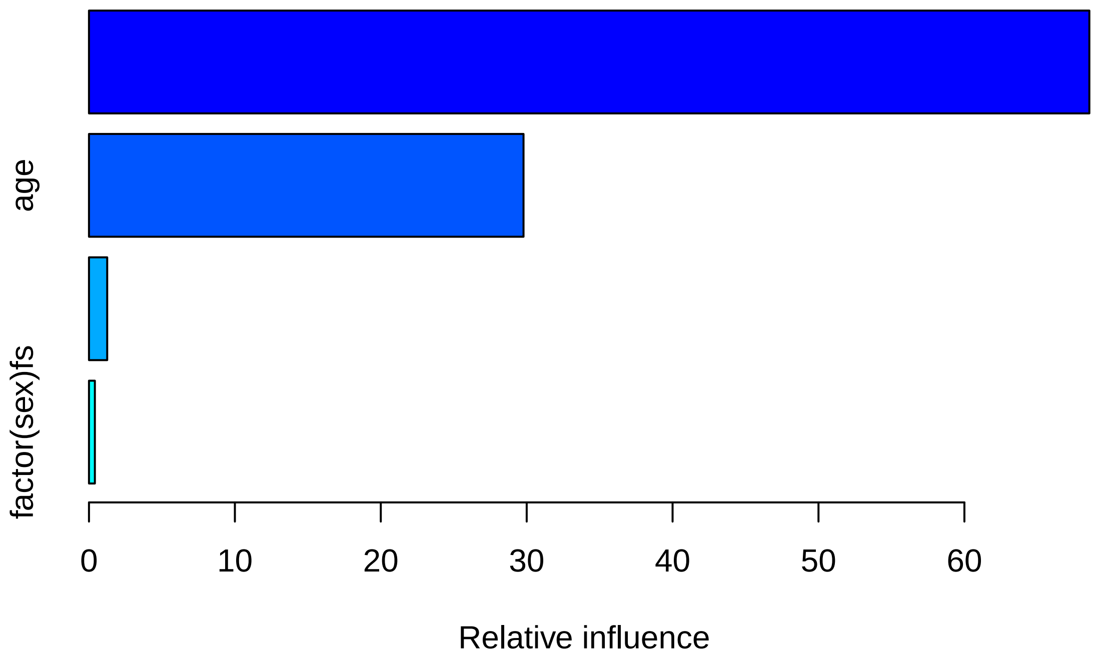


```
## Confusion Matrix and Statistics
##
##            Reference
```

```
## Prediction    benign malignant
##   benign    27.46988  18.27309
##   malignant 18.98260  35.27443
##
##                Accuracy : 0.6274
##                  95% CI : (NA, NA)
##     No Information Rate : NA
##     P-Value [Acc > NIR] : NA
##
##                   Kappa : 0.2504
##
##  Mcnemar's Test P-Value : 0.962
##
##             Sensitivity : 0.6588
##             Specificity : 0.5914
##          Pos Pred Value : 0.6501
##          Neg Pred Value : 0.6005
##              Prevalence : 0.5355
##          Detection Rate : 0.3527
##    Detection Prevalence : 0.5426
##       Balanced Accuracy : 0.6251
##
##        'Positive' Class : malignant
##
## AUC inner 0.662277
##
## Testing summary
```

```
## Setting levels: control = benign, case = malignant
## Setting direction: controls < cases
```

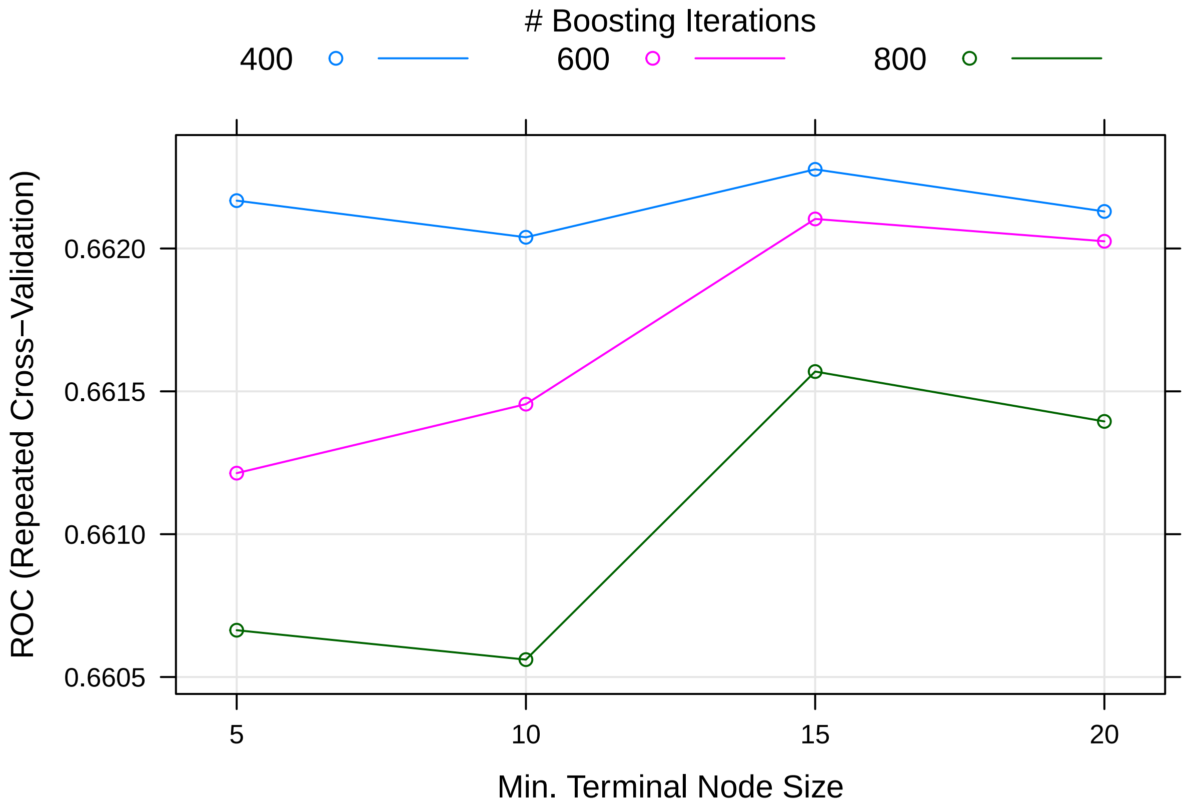


```
##
## Call:
## roc.default(response = test$twolevels, predictor = prob_predictions,     auc = TRUE, ci = TRUE)
##
## Data: prob_predictions in 173 controls (test$twolevels benign) < 199 cases (test$twolevels malignant
## Area under the curve: 0.6905
## 95% CI: 0.6373-0.7438 (DeLong)
## Confusion Matrix and Statistics
##
##            Reference
## Prediction  benign malignant
##   benign        94        60
##   malignant     79       139
##
##                Accuracy : 0.6263
##                  95% CI : (0.575, 0.6757)
##     No Information Rate : 0.5349
##     P-Value [Acc > NIR] : 0.0002283
##
##                   Kappa : 0.2436
##
##  Mcnemar's Test P-Value : 0.1268257
##
##             Sensitivity : 0.6985
##             Specificity : 0.5434
##          Pos Pred Value : 0.6376
```

```
##          Neg Pred Value : 0.6104
##              Prevalence : 0.5349
##          Detection Rate : 0.3737
##    Detection Prevalence : 0.5860
##       Balanced Accuracy : 0.6209
##
##        'Positive' Class : malignant
##
## ================================================================
## Fold  3
## Training summary
```

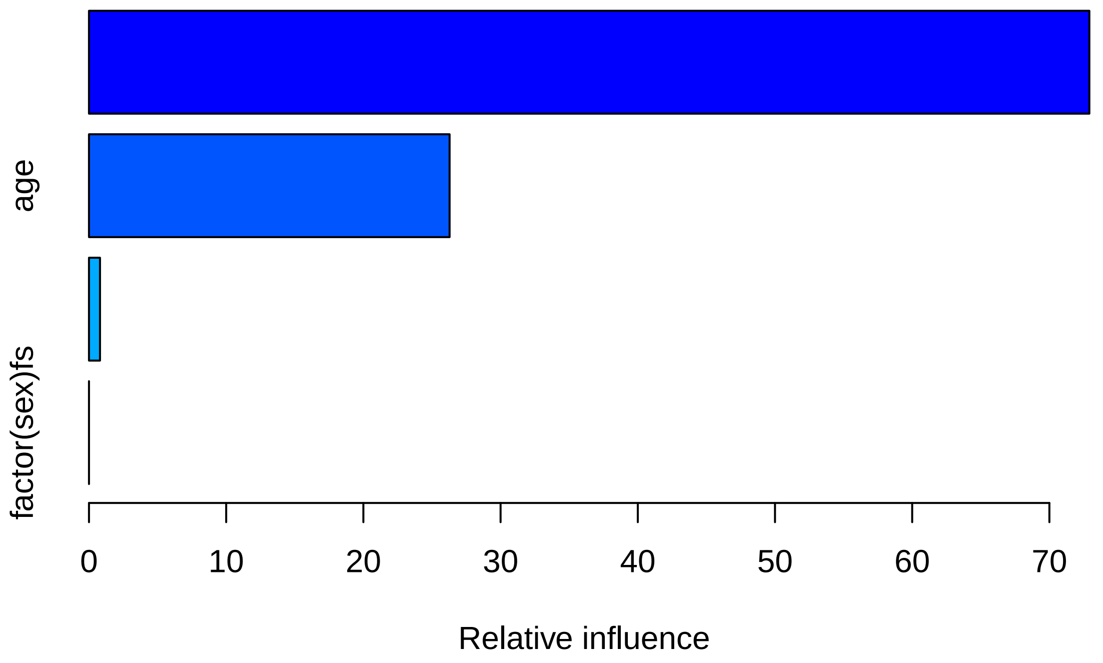


```
## Confusion Matrix and Statistics
##
##            Reference
## Prediction    benign malignant
##   benign    28.25184  19.20965
##   malignant 18.23175  34.30676
##
##                Accuracy : 0.6256
##                  95% CI : (NA, NA)
##     No Information Rate : NA
##     P-Value [Acc > NIR] : NA
##
##                   Kappa : 0.2485
```

```
##
##  Mcnemar's Test P-Value : 0.9971
##
##             Sensitivity : 0.6411
##             Specificity : 0.6078
##          Pos Pred Value : 0.6530
##          Neg Pred Value : 0.5953
##              Prevalence : 0.5352
##          Detection Rate : 0.3431
##    Detection Prevalence : 0.5254
##       Balanced Accuracy : 0.6244
##
##        'Positive' Class : malignant
##
## AUC inner 0.667094
##
## Testing summary
```

```
## Setting levels: control = benign, case = malignant
## Setting direction: controls < cases
```

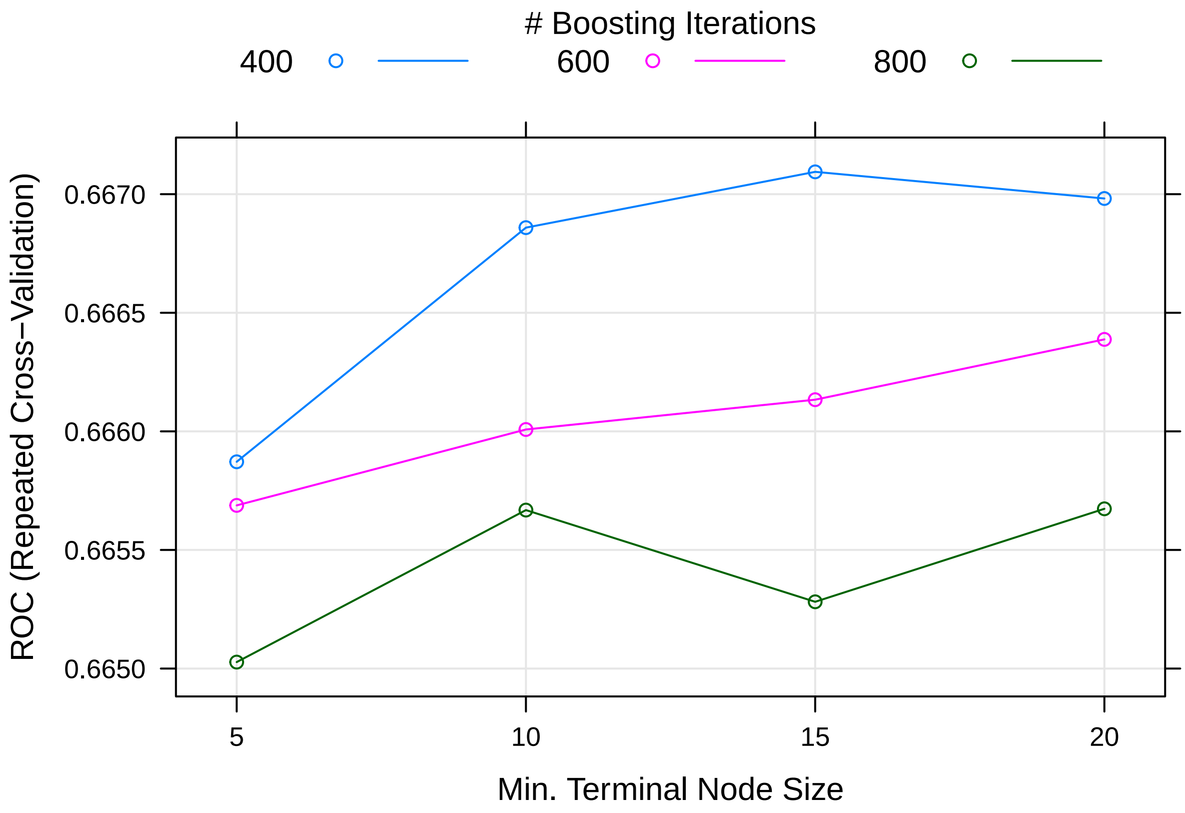


```
##
## Call:
## roc.default(response = test$twolevels, predictor = prob_predictions,     auc = TRUE, ci = TRUE)
##
```

```
## Data: prob_predictions in 173 controls (test$twolevels benign) < 200 cases (test$twolevels malignant)
## Area under the curve: 0.6665
## 95% CI: 0.6115-0.7216 (DeLong)
## Confusion Matrix and Statistics
##
##            Reference
## Prediction  benign malignant
##   benign       116        82
##   malignant     57       118
##
##                Accuracy : 0.6273
##                  95% CI : (0.5761, 0.6766)
##     No Information Rate : 0.5362
##     P-Value [Acc > NIR] : 0.0002315
##
##                   Kappa : 0.258
##
##  Mcnemar's Test P-Value : 0.0417852
##
##             Sensitivity : 0.5900
##             Specificity : 0.6705
##          Pos Pred Value : 0.6743
##          Neg Pred Value : 0.5859
##              Prevalence : 0.5362
##          Detection Rate : 0.3164
##    Detection Prevalence : 0.4692
##       Balanced Accuracy : 0.6303
##
##        'Positive' Class : malignant
##
## ================================================================
## Fold  4
## Training summary
```

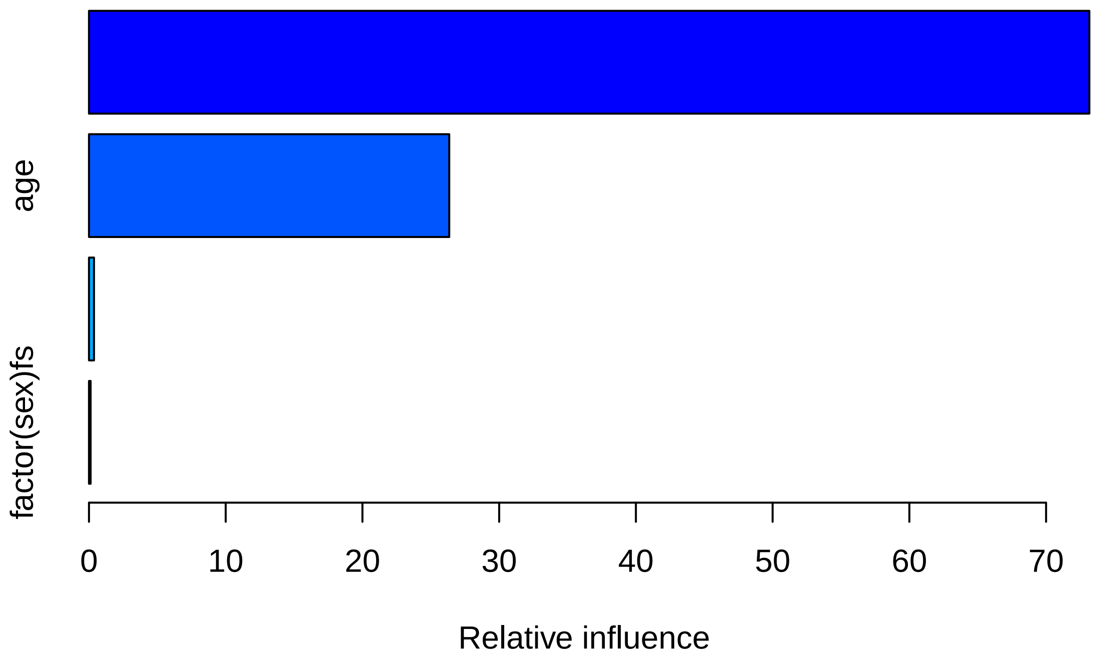


```
## Confusion Matrix and Statistics
##
##            Reference
## Prediction    benign malignant
##   benign    27.75620  19.19625
##   malignant 18.72739  34.32016
##
##                Accuracy : 0.6208
##                  95% CI : (NA, NA)
##     No Information Rate : NA
##     P-Value [Acc > NIR] : NA
##
##                   Kappa : 0.2383
##
##  Mcnemar's Test P-Value : 0.9313
##
##             Sensitivity : 0.6413
##             Specificity : 0.5971
##          Pos Pred Value : 0.6470
##          Neg Pred Value : 0.5912
##              Prevalence : 0.5352
##          Detection Rate : 0.3432
##    Detection Prevalence : 0.5305
##       Balanced Accuracy : 0.6192
##
##        'Positive' Class : malignant
```

```
##
## AUC inner 0.659870
##
## Testing summary

## Setting levels: control = benign, case = malignant
## Setting direction: controls < cases
```

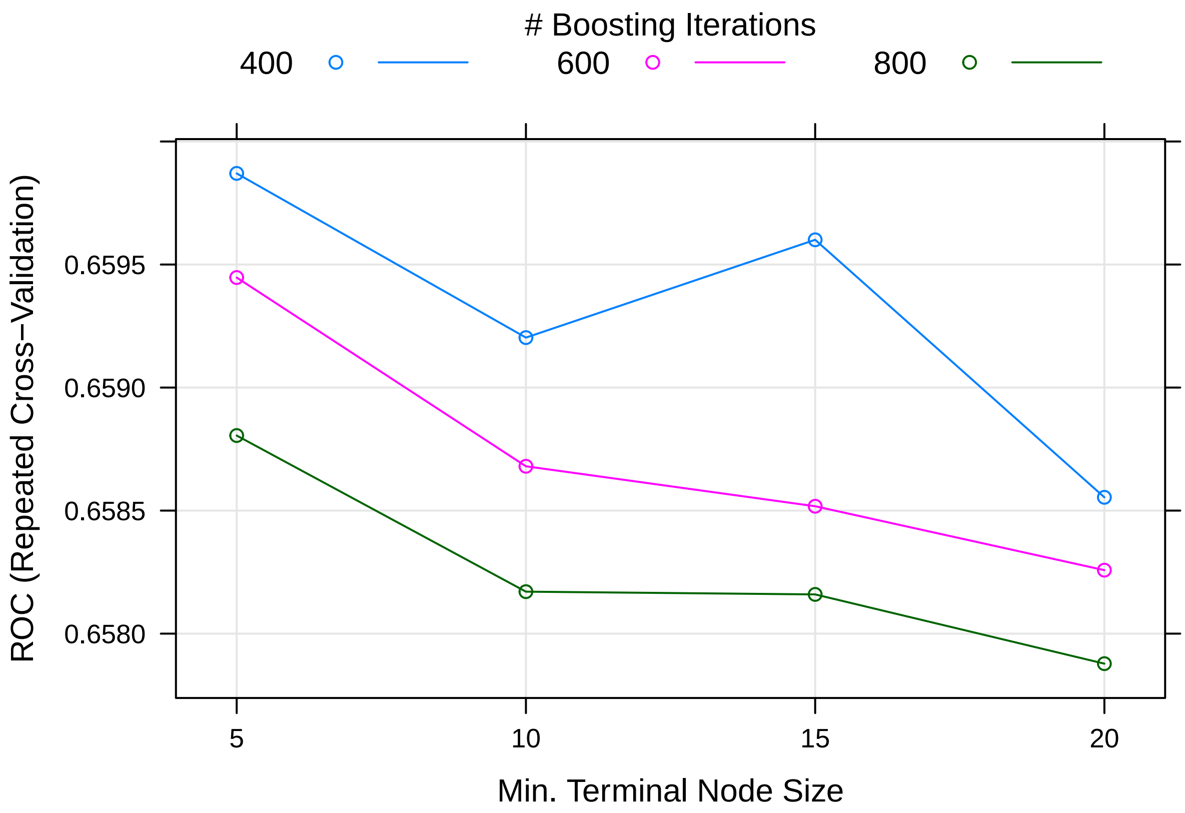


```
##
## Call:
## roc.default(response = test$twolevels, predictor = prob_predictions,     auc = TRUE, ci = TRUE)
##
## Data: prob_predictions in 173 controls (test$twolevels benign) < 200 cases (test$twolevels malignant
## Area under the curve: 0.6856
## 95% CI: 0.6322-0.7391 (DeLong)
## Confusion Matrix and Statistics
##
##            Reference
## Prediction  benign malignant
##   benign        99        67
##   malignant     74       133
##
##                Accuracy : 0.622
##                  95% CI : (0.5706, 0.6714)
##     No Information Rate : 0.5362
```

```
##     P-Value [Acc > NIR] : 0.0005005
##
##                   Kappa : 0.2379
##
##  Mcnemar's Test P-Value : 0.6133544
##
##             Sensitivity : 0.6650
##             Specificity : 0.5723
##          Pos Pred Value : 0.6425
##          Neg Pred Value : 0.5964
##              Prevalence : 0.5362
##          Detection Rate : 0.3566
##    Detection Prevalence : 0.5550
##       Balanced Accuracy : 0.6186
##
##        'Positive' Class : malignant
##
## =================================================================
## Fold  5
## Training summary
```

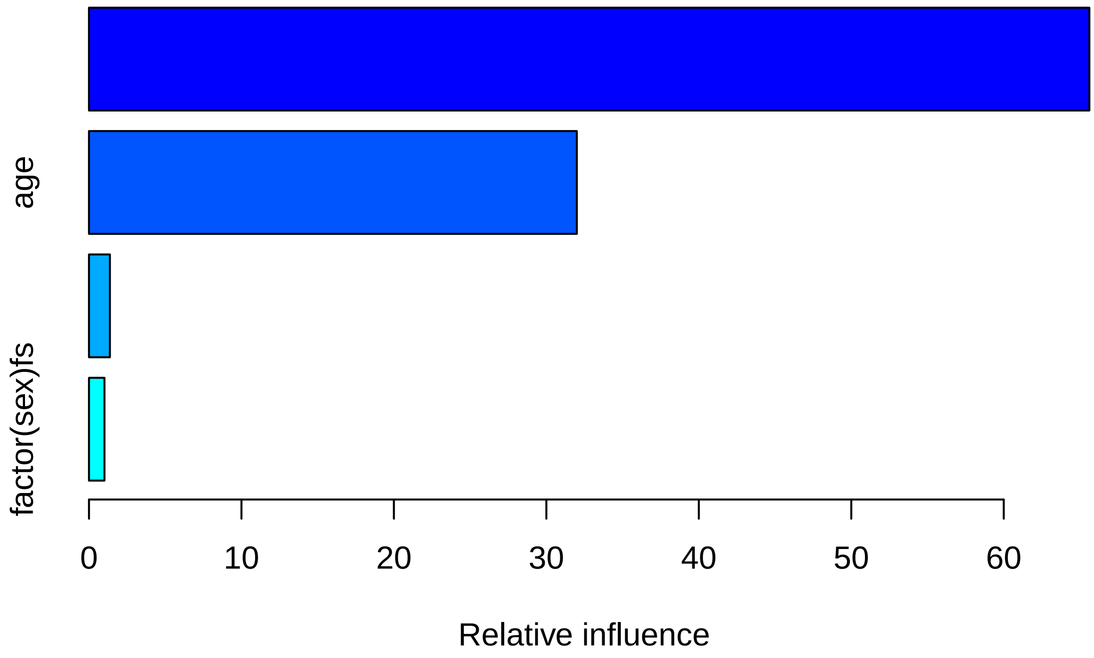


```
## Confusion Matrix and Statistics
##
##            Reference
## Prediction    benign malignant
```

```
##   benign    26.78284  18.24397
##   malignant 19.66488  35.30831
##
##                Accuracy : 0.6209
##                  95% CI : (NA, NA)
##     No Information Rate : NA
##     P-Value [Acc > NIR] : NA
##
##                   Kappa : 0.2364
##
##  Mcnemar's Test P-Value : 0.9455
##
##             Sensitivity : 0.6593
##             Specificity : 0.5766
##          Pos Pred Value : 0.6423
##          Neg Pred Value : 0.5948
##              Prevalence : 0.5355
##          Detection Rate : 0.3531
##    Detection Prevalence : 0.5497
##       Balanced Accuracy : 0.6180
##
##        'Positive' Class : malignant
##
## AUC inner 0.672070
##
## Testing summary
```

```
## Setting levels: control = benign, case = malignant
## Setting direction: controls < cases
```

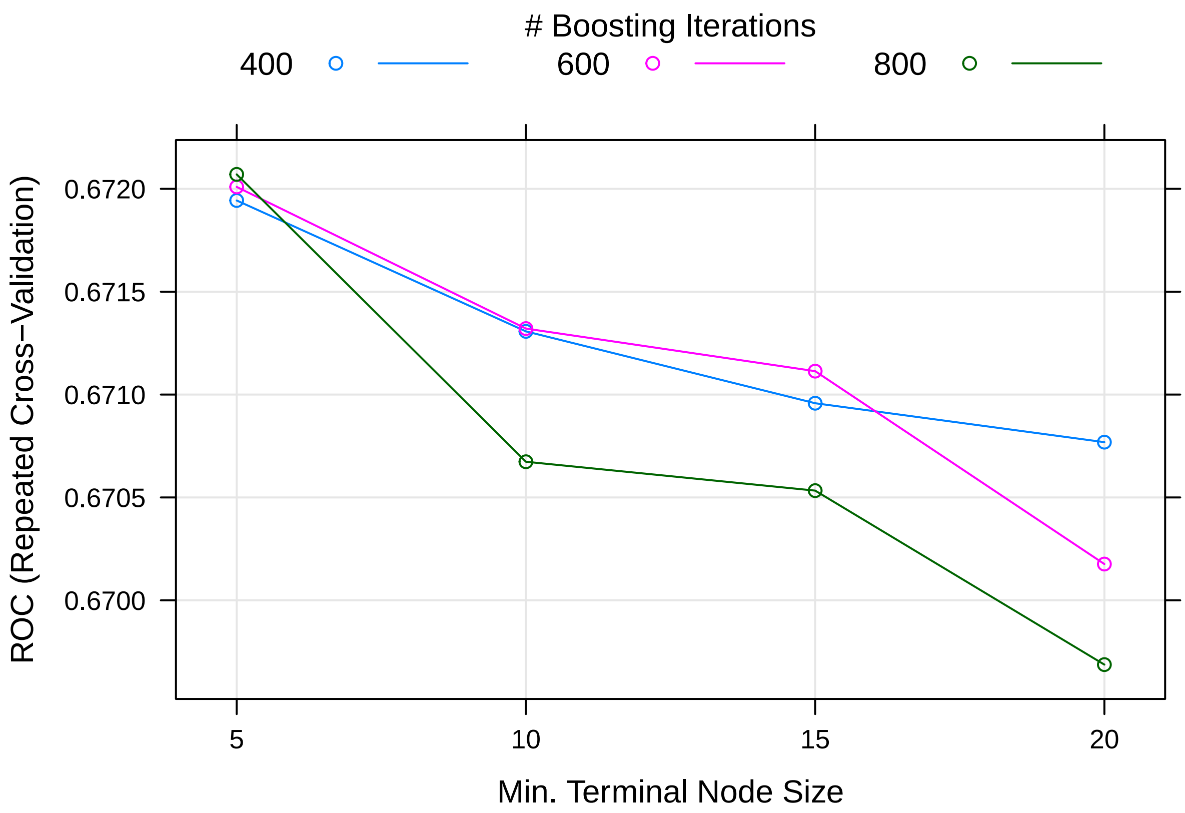


```
##
## Call:
## roc.default(response = test$twolevels, predictor = prob_predictions,     auc = TRUE, ci = TRUE)
##
## Data: prob_predictions in 174 controls (test$twolevels benign) < 200 cases (test$twolevels malignant
## Area under the curve: 0.648
## 95% CI: 0.5921-0.7038 (DeLong)
## Confusion Matrix and Statistics
##
##            Reference
## Prediction  benign malignant
##   benign       101        64
##   malignant     73       136
##
##                Accuracy : 0.6337
##                  95% CI : (0.5826, 0.6826)
##     No Information Rate : 0.5348
##     P-Value [Acc > NIR] : 6.898e-05
##
##                   Kappa : 0.2613
##
##  Mcnemar's Test P-Value : 0.4943
##
##             Sensitivity : 0.6800
##             Specificity : 0.5805
##          Pos Pred Value : 0.6507
```

```
##          Neg Pred Value : 0.6121
##              Prevalence : 0.5348
##          Detection Rate : 0.3636
##    Detection Prevalence : 0.5588
##       Balanced Accuracy : 0.6302
##
##        'Positive' Class : malignant
##
## =================================================================
```

```
#saveRDS(fullGbmFit, file=paste0(savePrefix, "fullGbm.rds"))
```

```
getResults(confusionMatricesGbm)
```

```
## [1] "Total"
## Confusion Matrix and Statistics
##
##            Reference
## Prediction  benign malignant
##   benign       512       351
##   malignant    355       648
##
##                Accuracy : 0.6217
##                  95% CI : (0.5992, 0.6437)
##     No Information Rate : 0.5354
##     P-Value [Acc > NIR] : 3.224e-14
##
##                   Kappa : 0.2393
##
##  Mcnemar's Test P-Value : 0.9101
##
##             Sensitivity : 0.6486
##             Specificity : 0.5905
##          Pos Pred Value : 0.6461
##          Neg Pred Value : 0.5933
##              Prevalence : 0.5354
##          Detection Rate : 0.3473
##    Detection Prevalence : 0.5375
##       Balanced Accuracy : 0.6196
##
##        'Positive' Class : malignant
##
## [1] "Average"
## Confusion Matrix and Statistics
##
##            Reference
## Prediction  benign malignant
##   benign       102        70
##   malignant     71       130
##
##                Accuracy : 0.622
##                  95% CI : (0.5706, 0.6714)
##     No Information Rate : 0.5362
```

```
##     P-Value [Acc > NIR] : 0.0005005
##
##                   Kappa : 0.2397
##
##  Mcnemar's Test P-Value : 1.0000000
##
##             Sensitivity : 0.6500
##             Specificity : 0.5896
##          Pos Pred Value : 0.6468
##          Neg Pred Value : 0.5930
##              Prevalence : 0.5362
##          Detection Rate : 0.3485
##    Detection Prevalence : 0.5389
##       Balanced Accuracy : 0.6198
##
##        'Positive' Class : malignant
##
## [1] "Single averages"
##
##  One Sample t-test
##
## data:  overall$Accuracy
## t = 103.92, df = 4, p-value = 5.142e-08
## alternative hypothesis: true mean is not equal to 0
## 95 percent confidence interval:
##  0.6050497 0.6382679
## sample estimates:
## mean of x
## 0.6216588
##
##
##  One Sample t-test
##
## data:  overall$`Balanced Accuracy`
## t = 105.38, df = 4, p-value = 4.862e-08
## alternative hypothesis: true mean is not equal to 0
## 95 percent confidence interval:
##  0.6033039 0.6359534
## sample estimates:
## mean of x
## 0.6196286
##
##
##  One Sample t-test
##
## data:  overall$`Pos Pred Value`
## t = 83.669, df = 4, p-value = 1.223e-07
## alternative hypothesis: true mean is not equal to 0
## 95 percent confidence interval:
##  0.6253360 0.6682624
## sample estimates:
## mean of x
## 0.6467992
##
```

```
##
##  One Sample t-test
##
## data:  overall$`Neg Pred Value`
## t = 70.636, df = 4, p-value = 2.407e-07
## alternative hypothesis: true mean is not equal to 0
## 95 percent confidence interval:
##  0.5709252 0.6176434
## sample estimates:
## mean of x
## 0.5942843
```

```r
print('Mean AUCs train')
```

```
## [1] "Mean AUCs train"
```

```r
t.test(unlist(gbmaucsTrain))
```

```
##
##  One Sample t-test
##
## data:  unlist(gbmaucsTrain)
## t = 276.27, df = 4, p-value = 1.03e-09
## alternative hypothesis: true mean is not equal to 0
## 95 percent confidence interval:
##  0.6598282 0.6732253
## sample estimates:
## mean of x
## 0.6665268
```

```r
print('Mean AUCs')
```

```
## [1] "Mean AUCs"
```

```r
t.test(unlist(gbmaucs))
```

```
##
##  One Sample t-test
##
## data:  unlist(gbmaucs)
## t = 70.11, df = 4, p-value = 2.48e-07
## alternative hypothesis: true mean is not equal to 0
## 95 percent confidence interval:
##  0.6404478 0.6932649
## sample estimates:
## mean of x
## 0.6668563
```

```r
print('Best tuning parameters')
```

```
## [1] "Best tuning parameters"
```

```r
for (mdl in fullGbmFit) {
  print(mdl$bestTune)
}
```

```
##   n.trees interaction.depth shrinkage n.minobsinnode
## 5     600                 1      0.01             10
##   n.trees interaction.depth shrinkage n.minobsinnode
## 7     400                 1      0.01             15
##   n.trees interaction.depth shrinkage n.minobsinnode
## 7     400                 1      0.01             15
##   n.trees interaction.depth shrinkage n.minobsinnode
## 1     400                 1      0.01              5
##   n.trees interaction.depth shrinkage n.minobsinnode
## 3     800                 1      0.01              5
```

```r
print(gbmsm[[1]])
```

```
##                                                    var    rel.inf
## new_macro_size                          new_macro_size 65.5406269
## age                                                age 33.3255543
## factor(sex)fs                            factor(sex)fs  0.8302293
## factor(breed_two)purebreed factor(breed_two)purebreed  0.3035895
```

```r
mat <- do.call(rbind, lapply(gbmsm, function(x) return(x$rel.inf)))
apply(mat, 2, t.test)
```

```
## [[1]]
##
##  One Sample t-test
##
## data:  newX[, i]
## t = 41.282, df = 4, p-value = 2.058e-06
## alternative hypothesis: true mean is not equal to 0
## 95 percent confidence interval:
##  64.50836 73.81109
## sample estimates:
## mean of x
##  69.15973
##
##
## [[2]]
##
##  One Sample t-test
##
## data:  newX[, i]
## t = 20.585, df = 4, p-value = 3.29e-05
## alternative hypothesis: true mean is not equal to 0
## 95 percent confidence interval:
##  25.56279 33.53351
## sample estimates:
## mean of x
##  29.54815
```

```
##
##
## [[3]]
##
##  One Sample t-test
##
## data:  newX[, i]
## t = 5.1652, df = 4, p-value = 0.006674
## alternative hypothesis: true mean is not equal to 0
## 95 percent confidence interval:
##  0.4272973 1.4205964
## sample estimates:
## mean of x
## 0.9239468
##
##
## [[4]]
##
##  One Sample t-test
##
## data:  newX[, i]
## t = 2.0899, df = 4, p-value = 0.1048
## alternative hypothesis: true mean is not equal to 0
## 95 percent confidence interval:
##  -0.1209579  0.8573223
## sample estimates:
## mean of x
## 0.3681822
```

```
#print(summary(fullGbmFit[[1]]))
#plot(fullGbmFit[[1]])#, metric='ROC')
```

Models comparison

```
for (i in 1:outerFolds) {
  cat('fold', i)
  print(compare_models(fullGlmFit[[i]], fullGbmFit[[i]], 'ROC'))
}
```

```
## fold 1
##  One Sample t-test
##
## data:  x
## t = -4.2236, df = 49, p-value = 0.000104
## alternative hypothesis: true mean is not equal to 0
## 95 percent confidence interval:
##  -0.012750824 -0.004529106
## sample estimates:
##    mean of x
## -0.008639965
##
```

```
## fold 2
##  One Sample t-test
##
## data:  x
## t = -4.795, df = 49, p-value = 1.557e-05
## alternative hypothesis: true mean is not equal to 0
## 95 percent confidence interval:
##  -0.016006011 -0.006552012
## sample estimates:
##   mean of x
## -0.01127901
##
## fold 3
##  One Sample t-test
##
## data:  x
## t = 1.4118, df = 49, p-value = 0.1643
## alternative hypothesis: true mean is not equal to 0
## 95 percent confidence interval:
##  -0.001169448  0.006693389
## sample estimates:
##   mean of x
## 0.002761971
##
## fold 4
##  One Sample t-test
##
## data:  x
## t = -2.5579, df = 49, p-value = 0.01367
## alternative hypothesis: true mean is not equal to 0
## 95 percent confidence interval:
##  -0.010310143 -0.001237723
## sample estimates:
##    mean of x
## -0.005773933
##
## fold 5
##  One Sample t-test
##
## data:  x
## t = -2.2296, df = 49, p-value = 0.03039
## alternative hypothesis: true mean is not equal to 0
## 95 percent confidence interval:
##  -0.0076980747 -0.0003994657
## sample estimates:
##   mean of x
## -0.00404877
```

```
## R version 3.6.3 (2020-02-29)
## Platform: x86_64-w64-mingw32/x64 (64-bit)
## Running under: Windows 10 x64 (build 19041)
##
## Matrix products: default
##
```

```
## locale:
## [1] LC_COLLATE=Italian_Italy.1252  LC_CTYPE=Italian_Italy.1252
## [3] LC_MONETARY=Italian_Italy.1252 LC_NUMERIC=C
## [5] LC_TIME=Italian_Italy.1252
##
## attached base packages:
## [1] stats     graphics  grDevices utils     datasets  methods   base
##
## other attached packages:
## [1] dplyr_0.8.5     pROC_1.16.1     caret_6.0-85    ggplot2_3.3.0
## [5] lattice_0.20-38 pacman_0.5.1
##
## loaded via a namespace (and not attached):
##  [1] gbm_2.1.5            tidyselect_1.0.0     xfun_0.12
##  [4] purrr_0.3.3          reshape2_1.4.3       splines_3.6.3
##  [7] vctrs_0.2.4          colorspace_1.4-1     generics_0.0.2
## [10] stats4_3.6.3         htmltools_0.4.0      yaml_2.2.1
## [13] survival_3.1-8       prodlim_2019.11.13   rlang_0.4.5
## [16] e1071_1.7-3          ModelMetrics_1.2.2.1 pillar_1.4.3
## [19] glue_1.3.2           withr_2.1.2          foreach_1.4.8
## [22] lifecycle_0.2.0      plyr_1.8.6           lava_1.6.7
## [25] stringr_1.4.0        timeDate_3043.102    munsell_0.5.0
## [28] gtable_0.3.0         recipes_0.1.9        codetools_0.2-16
## [31] evaluate_0.14        knitr_1.28           class_7.3-15
## [34] Rcpp_1.0.3           scales_1.1.0         ipred_0.9-9
## [37] gridExtra_2.3        digest_0.6.25        stringi_1.4.6
## [40] grid_3.6.3           tools_3.6.3          magrittr_1.5
## [43] tibble_2.1.3         crayon_1.3.4         pkgconfig_2.0.3
## [46] MASS_7.3-51.5        Matrix_1.2-18        data.table_1.12.8
## [49] lubridate_1.7.4      gower_0.2.1          assertthat_0.2.1
## [52] rmarkdown_2.1        iterators_1.0.12     R6_2.4.1
## [55] rpart_4.1-15         nnet_7.3-12          nlme_3.1-144
## [58] compiler_3.6.3
```
